# What Is Wellbeing, and What Is Important for Wellbeing? Indigenous Voices from across Canada

**DOI:** 10.3390/ijerph20176656

**Published:** 2023-08-26

**Authors:** Stephen R. J. Tsuji, Aleksandra M. Zuk, Andrew Solomon, Ruby Edwards-Wheesk, Fatima Ahmed, Leonard J. S. Tsuji

**Affiliations:** 1 Department of Physical and Environmental Sciences, University of Toronto, Toronto, ON M1C 1A4, Canada; amz4@queensu.ca (A.M.Z.); fa.ahmed@utoronto.ca (F.A.); leonard.tsuji@utoronto.ca (L.J.S.T.); 2 School of Environmental Studies, Queen’s University, Kingston, ON K7L 3N6, Canada; 3 School of Nursing, Queen’s University, Kingston, ON K7L 3N6, Canada; 4 Fort Albany First Nation, Fort Albany, ON P0L 1H0, Canada; 5 Department of Health and Society, University of Toronto, Toronto, ON M1C 1A4, Canada

**Keywords:** First Nations, Métis, Inuit, Canada, Indigenous peoples’ perspectives of wellbeing, valued components of wellbeing, wellbeing, land and water, impact assessment, sustainability

## Abstract

Indigenous peoples’ perceptions of wellbeing differ from non-Indigenous constructs. Thus, it is imperative to recognize that Indigenous peoples will conceptualize wellbeing from their perspectives and set their own wellbeing priorities. In keeping with this viewpoint, the aims of the present study were to conceptualize wellbeing and determine what was (and is) important for wellbeing from Canadian Indigenous peoples’ perspectives. In this paper, we take a partnership approach based on the elements of respect, equity, and empowerment. One primary data source and two existing data sources were examined and analyzed thematically utilizing a combination approach of deductive and inductive coding. Indigenous leadership and organizations viewed wellbeing holistically and conceptualized wellbeing multidimensionally. From across Canada, wellbeing was communicated as physical, economic, political, social, and cultural. The scaling of wellbeing represented a collectivist perspective, and land was the connecting thread between all types of wellbeing, being a place to practice cultural traditions, reassert one’s Indigenous identity, find solace, and pass on Indigenous knowledge and languages. Although wellbeing was discussed in the context of the individual, family, community, and nation, wellbeing was most often discussed at the cultural level by regional and national Indigenous leadership and organizations. Even in acknowledging the great cultural diversity among Canadian Indigenous nations, four concordant themes were identified regionally and nationally, with respect to what was important for cultural wellbeing: land and water, sustainability, and inherent obligations; being on the land, and indigenous languages and knowledge systems; sustainable development; and meaningful involvement in decision-making, and free, prior, and informed consent. Taking into account these themes is foundational for any interaction with Indigenous peoples, especially in the context of land, culture, and development. There needs to be a new beginning on the journey to reconciliation with land and cultural wellbeing at the forefront.

## 1. Introduction

Worldwide, wellbeing indicators have been used for approximately 50 years by governments to measure the progress of nations [1]; however, the concept of wellbeing is not well defined and often used interchangeably with the concepts of health, quality of life (e.g., life satisfaction), and happiness [1,2]. Nonetheless, wellbeing has been reported to exist in two dimensions: the objective and the subjective [3]. Historically, singular measures (e.g., Gross Domestic Product (GDP)) were used to measure objective wellbeing [4,5], but it was recognized that there was a need to move beyond the use of the GDP and incorporate data on social and environmental factors to better measure wellbeing and progress [1]. Thus, worldwide composite measures were developed, such as the Human Development Index (HDI) used by the United Nations (UN) [6], the Organization for Economic Cooperation and Development’s Better Life Initiative [7], and the UN’s Sustainable Development Goals [8]. Measures of health appear as a component of wellbeing in all the above composite measures [7,8], while a variety of other variables, for instance, environmental quality and subjective wellbeing, appear in some.

In Canada, the Registered Indian and Inuit Human Development Index [9] and Community Well-Being Index [10] were developed by the Government of Canada to track the progress of the health and wellbeing of Indigenous peoples in Canada [9,10]. There has been criticism of these composite indices because of the lack of inclusion of cultural factors [11]; thus, the adaptation of the HDI was viewed as inappropriate and/or problematic [11]. Similarly, in Australia, composite indices without Indigenous cultural variables were said to be unreliable and/or inappropriate in the context of assessing Indigenous wellbeing [12]. As stressed by Dockery [13], Indigenous cultures must be considered part of the solution to improve wellbeing rather than the problem. The collection of Indigenous peoples’ data must do more than just servicing government needs [14]—such as assessing closing the gap initiatives worldwide with respect to Indigenous and non-Indigenous peoples’ health [15,16], a deficit model approach [17]. There must be a transition to supporting Indigenous peoples’ development agendas, aspirations [14], and Indigenous perspectives of wellbeing [2], a strengths-based approach [18,19]. For example, one exploratory study with two First Nations in Canada included a base questionnaire about satisfaction with six domains of wellbeing (i.e., education, employment, health, housing, income, and social–cultural), while a seventh domain (i.e., land use) was incorporated into the study after being identified by the Indigenous peoples [20].

In contrast to the objective-quantitative wellbeing dimension, the subjective dimension of wellbeing has been reported to be composed of an individual’s experience of their life [21]. It is often assumed that subjective wellbeing is restricted to the measurement of happiness, but subjective wellbeing may include measures of eudaimonia (i.e., meaning and/or purpose in life [21]). In Canada, the Canadian Index of Wellbeing was launched to measure subjective wellbeing, but this composite measure was not informed by Canada’s Indigenous peoples, while non-Indigenous Canadians were consulted [22,23]. As stated by the UN Technical Workshop on Indigenous Peoples and Indicators of Well-Being, extensive dialogue with Indigenous peoples, communities, and organizations is required in order to properly describe Indigenous views of wellbeing [24]. This is especially true considering that Indigenous peoples’ perceptions of wellbeing differ and extend beyond government-constructed frameworks of wellbeing [17,25], creating tension between the two groups due to different worldviews [2]. As asserted by the United Nations (UN) Permanent Forum on Indigenous Issues [24] (p. 10): “*The United Nations system and states should recognize that Indigenous peoples will define their own understandings and visions of well-being from which indicators will be identified, and include the full participation of Indigenous peoples in the development of these indicators.*” Indeed, First Nations peoples want to conceptualize what wellbeing means for them and their future [11]—and at the local level, Indigenous communities have started the process of setting their own wellbeing priorities [18]—but there is a need to first conceptualize wellbeing from an Indigenous perspective, and identify valued aspects of wellbeing [18]. Accordingly, the present study objectives are threefold: (1) to conceptualize wellbeing from a community-level (northern Ontario, Canada) First Nations Elders’ perspective, and identify valued aspects of wellbeing to help inform local policy and community-based wellbeing programs; (2) to explore what is important for wellbeing from a regional-level (northern Ontario) First Nations’ perspective; and (3) to examine at a national-level, what is important for wellbeing from First Nations, Inuit, and Métis perspectives.

## 2. Methods

The present project builds upon our team’s previous work with Fort Albany First Nation—during Chief A. Solomon’s (AS) several terms in office—that focused on treaties [26,27,28,29] and resource development (e.g., diamond mining [30,31,32]; hydroelectric development [33,34]). It was the idea of AS to bring together “the real, unelected leaders” of their people in a forum where they could identify and discuss the core elements needed to sustain Cree culture. This concept of the core elements that need to be sustained to protect Cree culture with respect to resource development arose during a Mushkegowuk Tribal Council forum on proposed hydroelectric development in the region, as a question was raised about what would happen if the Cree did not want any development on (or further development on) a river, such as the Albany River. In other words, what if the Albany River was identified by the First Nations people as a core element of Cree culture, whereby any further hydroelectric development on the Albany River would irreparably impact Cree culture? This is a fundamentally different question and approach to resource development espoused by mainstream planning. Most non-Indigenous societies view the environment as just “space” to be exploited for material gain rather than something with spirit to be revered, stewarded, and sustained.

### 2.1. The Study Area

**Figure 1:**
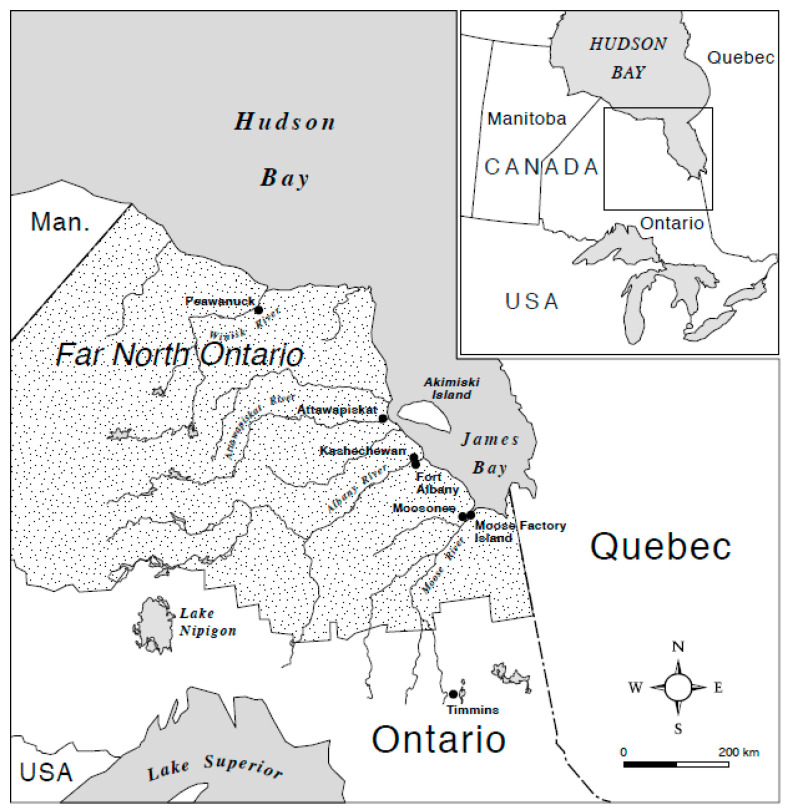
Fort Albany First Nation and the Far North region of Ontario (stippled area).

Fort Albany First Nation is an Omushkego Cree community located in the western James Bay region of subarctic Ontario, Canada (Figure 1). Ontario is the second largest Canadian province [35], and it is home to more Indigenous (First Nations, Inuit, and Métis) peoples than any other province [36]. There are 133 First Nations located throughout Ontario [37] and 13 distinct groups [36]. Northern Ontario and its Far North region (Figure 1) are home to 49 First Nations of Cree, Oji-Cree, and Ojibwe heritage; these First Nations and their Tribal Councils (i.e., regional First Nations’ organizations) comprise Nishnawbe Aski Nation (i.e., a supra-regional First Nations’ organizations).

**Figure 2:**
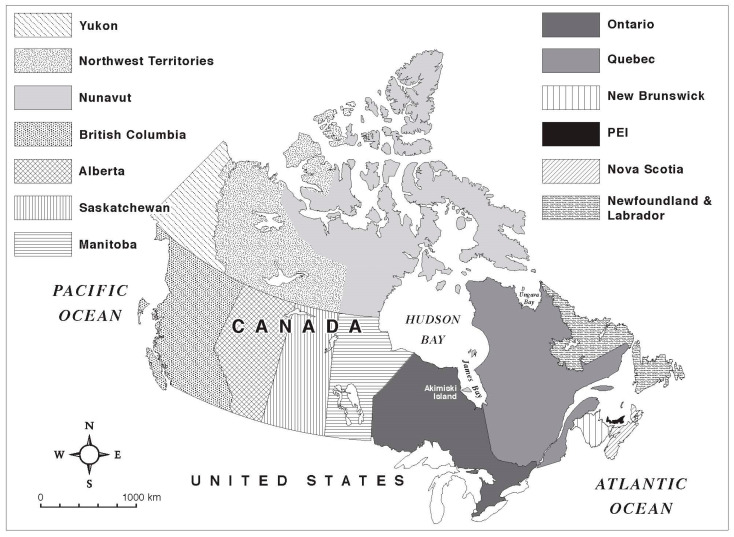
Canada’s ten provinces and three territories (i.e., Yukon, Northwest Territories, and Nunavut).

Canada is a federation of ten provinces and three territories (Figure 2); it has a land mass of ~9,984,670 km^2^, which makes it the second-largest country in the world [38]. The Indigenous population of Canada was recently estimated at 1,673,785 or ~4.6% of the total Canadian population [39,40]. First Nations peoples were reported to be the most numerous (977,230), then the Métis (587,545), and finally the Inuit (65,025) (note: people of multiple Indigenous identities numbered 21,310 people, while there were 22,670 individuals of other Indigenous ancestry) [39]. In Canada, Indigenous peoples have diverse cultures based on their relationships with their homelands [39]. First Nations peoples live in ~600 unique First Nations [39], but the majority of First Nations peoples live off-reserve (i.e., not in a First Nation [41]). Most Métis inhabit the western provinces of Canada and the Province of Ontario in an urban setting [39]. The majority of Inuit inhabit their homeland of Inuit Nunangat [39], composed of four regions: Inuvialuit; Nunavut; Nunavik; and Nunatsiavut [42].

### 2.2. Ethics

This project was initiated in partnership with Fort Albany First Nation following a community-based participatory approach utilized in past projects [43]; no formal ethics protocol exists for this community. Nonetheless, we followed a partnership framework that we had previously developed and used. In 2003, Fort Albany First Nation and our research team began formulating a partnership framework that, in the end, identified three essential elements that must be included to be considered a true partnership with First Nations: respect (e.g., the recognition of Indigenous knowledge systems as equal to western knowledge systems); equity (e.g., the sharing of resources); and empowerment (e.g., the sharing of power through means such as governance) [44]. This partnership framework guided the present project. For example, leadership was shared in the project with members of the research team being Indigenous (specifically, Omushkego Cree; AS, REW) and non-Indigenous (ST, AZ, FA, LT). Additionally, potential discipline bias was mitigated through the interdisciplinary composition of the research team, which included members with expertise in the social sciences and humanities (ST, AS, REW, LT), the health sciences (AZ, AS, REW, FA, LT), and the physical and natural sciences (AS, LT). Furthermore, an Indigenous Advisory Board was employed. Members of the Indigenous Advisory Board were involved throughout the formative stage of the proposed project and were directly involved throughout the implementation of the project, guiding the process. A set of questions was developed and informed by the project’s Indigenous Advisory Board. This approach ensured that the questions were culturally appropriate and relevant. The appropriateness of the questions was finalized through pre-testing with a small group. The board members were the gatekeepers of their intellectual property and ensured community needs, customs, knowledge and experiences were valued and protected. Community customs and codes were built into the project’s approach—e.g., community leadership consent prior to individual oral consent for participants—and Cree language translators were used when requested. Relationships were reciprocal, and there were benefits for all involved.

All project activities were in accordance with the ethical standards of the University of Toronto, Ontario, Canada, and approved by its Research Ethics Board (Protocol Reference # 33374). Informed consent, either in Cree or English, was given by all Elders prior to the start of their participation.

### 2.3. Data

#### 2.3.1. Fort Albany First Nation, Ontario, Canada

First Nations reserve lands were created during the treaty-making process in Canada, being artificial constructs [28]. First Nations are governed by a Chief and Council (Band Council)—a treaty construct—and/or by Hereditary Chiefs. For the present study, a community Elders list identified potential participants. All Elders on the list were approached to participate. From September 2017 to February 2019, semi-directed interviews in Cree and/or English were conducted with participating Elders using the conversational method [45]. Interview questions were translated into Cree (or Cree syllabics), dependent on the Elder’s preference. Elders participated either individually or as a couple. The questions asked relevant to the present study were as follows: 1. What is wellbeing? 2. What is cultural wellbeing? 3. What is important for cultural wellbeing? Responses were digitally recorded, or if the participant preferred, written comments were accepted. Digital recordings in English were transcribed verbatim, while oral or written responses in Cree were translated into English. Of the 24 people on the Elders list, nine participated (8 males and one female), while the others were unable to participate for various reasons (e.g., declining health, memory attrition), and five chose not to participate and gave no reason. Of the nine participants, seven gave individual responses, while one couple gave a joint response.

#### 2.3.2. Northern Ontario, Canada

In Canada, governance is through a centralized (or federal) government and regional (or provincial) government [46]. The Province of Ontario employs a unicameral legislative branch that introduces, debates, and passes Bills, as well as amends Acts (i.e., statutes or laws) [47]. Of relevance, the Ontario legislature has an ethical, fiduciary responsibility to consult with all Ontarians (including Indigenous peoples) during the law-making process, when a Bill is being considered by the legislature, and before the Bill becomes an Act [48,49]. It should be noted the ‘duty to consult’ Indigenous peoples of Canada is never triggered during the law-making process—because only an ethical, fiduciary responsibility to consult with Indigenous people exists [49]—there is no legal fiduciary responsibility during the Bill-to-Act process that occurs within the legislative branch of government [50].

In Ontario and Canada, the government Bill-to-Act process is based on the Westminster model [48,51], whereby consultation with the public, including Indigenous peoples and organizations, can be realized through public hearings that typically occur after First Reading of a Bill, but before Second Reading in parliament [48,49,52,53,54]. At the Standing Committee stage, with public hearings, debate and consideration for Bill amendments occurs [48]. During the committee hearings, oral presentations and discussions are recorded, with verbatim transcripts of the legislature debates being produced.

In order to present a northern Ontarian First Nations perspective on what is important for cultural wellbeing or the valued components of cultural wellbeing, existing data were collected. Existing data were in the form of Hansard verbatim transcripts of the Government of Ontario’s Standing Committee on General Government public hearings for Bill 173 (i.e., the *Mining Amendment Act* [55]) and Bill 191 (i.e., *the Far North Act* [56]). It should be mentioned that the Hansard verbatim transcripts for public hearings with respect to the *Ontario Environmental Assessment Act*—which was contained in the omnibus bill (i.e., Bill 197) entitled the *COVID-19 Economic Recovery Act* [57]—would also have been appropriate for the present study [57]. Unfortunately, there were no committee meeting hearings for the *COVID-19 Economic Recovery Act* [57] because this piece of legislation was passed quickly through the Ontario legislature in just 14 days [49,57]. For Bills 173 and 191, Hansard transcripts were read in their entirety.

#### 2.3.3. Canada

The Government of Canada utilizes a bicameral legislature that includes the elected House of Commons and the appointed Senate [51]. The Bill-to-Act process at the federal level follows the same general procedures as for the Government of Ontario, except that both chambers must pass a Bill before it becomes an Act [51]. In addition to public hearings being recorded and verbatim transcripts produced, written submissions can be made during the federal public hearing process, and these submissions are made available to the public.

In order to illustrate Canadian Indigenous perspectives on what is important for cultural wellbeing (or the valued components of cultural wellbeing), existing data were collected in the context of Bill C-69 [58,59]. Existing data in the form of all written submissions to the Government of Canada’s Standing Committee on Environment and Sustainable Development were examined. Additionally, the Hansard verbatim transcripts of Bill C-69′s Standing Committee on Environment and Sustainable Development public hearings were examined. These two sources of existing data for 2018 provided insight into how Canadian Indigenous leadership and organizations viewed wellbeing and what was important for cultural wellbeing.

### 2.4. Data Analyses

Qualitative data from semi-directed interviews, submissions, and Hansard transcripts were analyzed utilizing a combination approach of deductive and inductive thematic coding [43,60]. The data were first deductively analyzed into themes [61], using a template organizing approach [60] whereby the questions asked provided the thematic framework. Subthemes to these main themes arose through inductive and iterative thematic coding that let the themes emerge from the data [43,62]. The first author was primarily responsible for analysis with formative discussions with the rest of the research team, and preliminary results were discussed with the Indigenous Advisory Board members. Findings were validated with a third of the participating Elders. The validation process was interrupted in 2020 and then discontinued due to a variety of reasons (e.g., the COVID-19 pandemic resulted in the closure of the borders of Fort Albany First Nation in the spring of 2020, for more than two years; and participant senescence).

## 3. Results

### 3.1. What Is Wellbeing?

#### 3.1.1. A Fort Albany First Nation Elders’ Perspective

Responses to this question were generally succinct, with wellbeing being described in a variety of ways related to physical and mental health, such as “*Someone who has no illness.*” (C5); “*[W]hen one is happy. When one is not sick. You are able to do whatever you want to do.*” (C4); “*You’re well…I am not living a good life right now because I go to the hospital every month almost.*” (C2); and “*Someone with healthy body and spirit, all things that are possessed inside everyone.*” (C3) Others described wellbeing in terms of having “*family when one is growing up*” (C6), their “*traditional land*” (C1), and “*going out*” on the land (C2). Going out on the land was described as being important to mental health and Indigenous identity:

“*For me wellbeing is like trying to look after your mental health, your identity as a native person. So trying to practice your cultural traditions, like going out on the land. So that you can pass it [Cree knowledge] onto the younger generations…So to me to keep that oral tradition. That is the way people were a long time ago. Oral passage of knowledge to young people. So that is how I came to know the things that I know today. That is how it was passed onto me. No books; I didn’t go to school. I was with Elders all the time. They just teach me. Sometimes they did not have to say anything. Just by example…that is how I learnt. That is how to be a better person for your whole self to be a whole person. Improve on your wellbeing, you have to work on it. You cannot be pushed into it; it is something you do of your own free will.*”

(C8)

Wellbeing was also mentioned in the context of balance and harmonious relationships, relatedness, and honouring inherent obligations:

“*Wellbeing, my understanding as a whole, in general, is the sense of the balance of the four components of our being: body, mind, spirit, and emotions. That is wellbeing. There has to be the balance in the context of wellbeing…And in the context of creation or the environment, all in one. Looking after what the Creator has provided for us, to respect and honouring what we have, where we live, and always be mindful of the environment. Like we don’t try to, like what is happening around the south: cutting the forests; damming the rivers; [and disrupting] the natural life of the environment. That [development] has to be in harmony with what the intent, the purpose that the Creator gave us in relation to all of creation…there are certain understanding of what people believe in…different races…need to live in harmony with them. To respect where they come from, their views. Not to put down people or individuals and to try to be respectful of what they believe in, and keep an open mind, and not always try to say: ‘ours is better than yours.’ None of that has to be part of the conversation. When you talk with someone, respect what they say. You can share that when you have discussions…always be mindful of how people are related to one another, related to the environment, with creation, all the components.*”

(C7)

In probing whether there were different types of wellbeing, one Elder addressed wellbeing in the context of scale:

“*Yeah there is different types of wellbeing. For example, here, we are so isolated from the rest of the world. For some people their worldview is not that wide especially for the younger people, here…They do not know what is going on out there…Some have a bigger worldview and they understand what is going on in the world. They understand that there are different races of people, that there are different Indigenous people in the world. That they are not the only Indigenous people in North America, just all over the world…so they understand that…they are not the only people that were suffering losing culture or language. Also, [they were] not only people working hard to revitalize their languages.*”

(C8)

#### 3.1.2. Northern Ontarian First Nations’ Perspectives

First Nations, Tribal Councils, and Nishnawbe Aski Nation were involved in the committee hearings for Bill 173 and Bill 191 to have their voices heard. Table S1 (see Supplementary Materials) presents the extent of involvement by First Nations political organizations in the hearings. No definition of wellbeing was found in committee hearing transcripts, nor were different types of wellbeing mentioned.

#### 3.1.3. Indigenous Perspectives from across Canada

First Nations, Inuit, and Métis political and Aboriginal organizations (community, regional, supra-regional, and national) from across Canada were involved in the written submission process and/or committee hearings for Bill C-69 to move their perspectives forward. Aboriginal is the term defined in the repatriated *Canadian Constitution Act*, *1982*, for Indigenous Peoples of Canada. Table S2 highlights the breadth of national coverage of Indigenous perspectives; conspicuous in their absence were northern Ontarian First Nations’ perspectives, except for those included in regional and national Indigenous organizations. No definition of wellbeing was presented in the submissions and related hearings for Bill C-69. However, different types of wellbeing were mentioned, such as economic wellbeing [63,64,65], political wellbeing [65], social wellbeing [64,65,66], and cultural wellbeing [65].

### 3.2. What Is Cultural Wellbeing?

#### **A Fort Albany First Nation Elders’ Perspective** 

For some Elders, cultural wellbeing was conceptualized briefly in the context of land (“*Where we are from,*” C1; “*resources to survive,*” C3), traditional pursuits (“*hunting, fishing*”; C2), traditional foods (“*In the past, since white man’s food were not consumed, people were healthy.*” C5), continuation of the “*Omuskego [Cree] language*” (C3), spirituality (“*Spiritual [is] part of it, as well body. Not only is worldly realm essential but your soul [is also important]*”; C6), and “*when people are doing well…not engaging in unhealthy activities such as drinking or drugs or other types of harm*” (C4). One Elder gave a more fulsome response:

“*For me, I guess I define it as what I do; I go out on the land. Sometimes I just spend time out there, before I go hunting. I usually go for a ride just to see what is out there…it also helps me to reconnect with my ancestors. We say that the wind and the trees when they make noises, they are our ancestors, spirits, talking to us. It is a good feeling to be out there. So that for me that is how I rejuvenate myself after a stressful week sometimes at work. You can be stressed out…at what is happening in your community, based on your social environment…We have right now people who are having social problems. Sometimes it is just people do not eat healthy because the way they are exposed to different types of environments. Like for example, drugs going around. People are exposed to that…become sick, and sad and depressed. Those are for the people that stay in the community, but for the people that go out on the land, it is much easier for those people to regain their strength to be in control, instead of someone else controlling.*”

(C8)

Lastly, an Elder mentioned cultural wellbeing in the context of inherent duties and harmonious relationships:

“*Cultural wellbeing [is], all that we were given when we first came to the face of the earth by the Creator, [everything] that the Creator gave us when we were born…[and] maturing into…a responsible adult. And doing what you are supposed to be doing–teaching–learning to teach is the intended purpose for us to live in harmony with us and all of creation…there is a way to take the negatives of what has happened to us and turn it into a positive. That would really help with harmony, culturally not forget that there is a purpose to our life that the Creator gave you.*”

(C7)

### 3.3. What Is Important for Cultural Wellbeing?

#### 3.3.1. A Fort Albany First Nations Elders’ Perspective

Several Elders identified the environment (C1) (C7) or, more specifically, Cree’s “*traditional land*” (C1) as being important for cultural wellbeing. The land and everything associated with the land was viewed as a gift: “*It [cultural wellbeing] was given to you by the spirit, your culture and all things on earth and creation so you can live and work properly.*” (C6) This gift of land and resources (C3) allowed for on-the-land traditional cultural activities (C2) (C8), such as spiritual pursuits (C6) (C8), and the procurement of traditional foods (C5), but with this gift came obligations that needed to be honoured to maintain the harmonious balance and interrelationships (C7). The Cree language (C3) (C8), Cree knowledge (C8), cultural traditions (C4), and the sharing of knowledge (C7) (C8) were identified as essential components necessary for cultural wellbeing.

One Elder forcibly espoused that:

“*The most important [thing for cultural wellbeing] is…for people to identify themselves as Native people. To teach their kids who they are…Sometimes I think people feel ashamed that they do not speak their language and then they blame themselves, because their children do not speak it. That they did not work hard enough to teach themselves to speak their language. Those are the challenges that the younger people face today—that they lost their language, and their children cannot speak their language…We need to educate our people on that, on why it is the way it is. They need to know their history.*”

(C8)

However, the challenge is:

“*We can’t do that [teach our own history] because the government controls the money [for education]. They only give us so much money for what they want us to learn. So, we have to fight to make changes to what they did to us. They imposed on us laws so that we do not have our own education, because the government says that this is how you are going to learn, even though we are on a reserve. You have to follow these regulations, or they will not give you the funding. So those are more challenges. Those are being worked on right now.*”

(C8)

Lastly, one Elder noted that Cree culture should not be considered static because “*Cree culture is changing.*” (C3)

#### 3.3.2. A Northern Ontarian First Nations’ Perspective

##### **Land and Water, Sustainability, and Inherent Obligations** 

The land was identified as being of utmost importance to cultural wellbeing in the context of First Nations peoples’ connection to it, stewardship of it, and sense of identity associated with it (Table S3). Specifically, Chief Jonathon Solomon of Kashechewan First Nation [67] (p. 954) eloquently states that

“*We live in the north. The land up north is our home. It’s our lifeline, it’s our bloodline of who we are. The land up north is not an untouched land. Our people, my ancestors, travelled that land. All over the area of my land, you can see sacred burial grounds, where my people died, where they lost their loved ones during the winter months. So, it’s not an untouched land; it’s not a land that has been discovered. We’ve been there for thousands and thousands of years. We were very nomadic people. We are still closely tied to the land. Like I said, that is our bloodline, our lifeline. Without land, we will [not] be Cree people of James Bay… Where there are footprints all over the place in my territory, that signifies that my people were out in the land.*”

The land provided sustenance to the First Nations peoples for millennia and continues to do so because the First Nations peoples, as stewards of the land, respected the environment and thought of the future (Table S3). The honouring of their inherent obligations was an important part of the First Nations’ culture that was not extinguished by colonialism (Table S3). In the words of Grand Chief Stan Beardy of Nishnawbe Aski Nation [68] (pp. 828–831):

“*The north is our homeland, and we govern and protect it through our inherent right, given to us by the Creator. Since time immemorial, our people have exercised our inherent right and protected the lands. That is why they are still in pristine condition. And we will continue to protect our lands for future generations…the Far North, it’s only First Nations people who live there. We have lived there for close to 10,000 years and we have preserved the natural environment up until now. We will continue to protect the natural environment.*”

##### **Being on the Land, and Indigenous Languages and Knowledge Systems** 

Being able to go on the land allows for cultural stories, history, laws, customs, and languages to be transmitted across and between generations (Table S3). Interestingly, typical measures of wellbeing, such as economic wealth and housing, were described as secondary to land, language, and culture from a First Nations’ perspective:

“*The majority of our members are living in poverty. Our community is cramped and on a little over two square miles of land and we have a significant housing crisis…Further, we are routinely evacuated from our community during break up [and flooding or threat of flooding], yet despite all this I believe we are one of the wealthiest First Nations in Canada. We still have our language, our culture and we are still able to go out on our land and to engage in our traditional aboriginal practices.*”

(Chief Theresa Hall of Attawapiskat First Nation) [69] (p. 981)

##### **Sustainable Development** 

It should be emphasized that First Nations are not against development per se, but the development must be sustainable from their perspective (Table S3). To the point, development to date in Ontario has irreparably damaged the environment, impacted First Nations’ culture, and has not been sustainable from their perspective (Table S3). As noted by Chief David Babin of Wahgoshig First Nation [70] (p. 955):

“*[W]e protect our lands. They’ve been protected for thousands of years. European people have come here, and look what they’ve developed; they’ve developed a land of disaster. They take all the revenues and whatever and leave, and leave us with nothing. Then we have to do the cleanup…Our people are getting sick from all these industries that are coming around our territory… I was talking about development with the hydro dams and the damage they’ve done. They washed away our graveyards into the lakes, and yet development still happens…Development, yes, but how much do we develop before we start…Destroying the lands, our rivers, our waters.*”

Nonetheless, development is not the only contributing factor that is keeping the First Nations’ peoples off the land and partaking in cultural activities. Protected parks act as barriers to cultural pursuits, and displacement off the land has negative consequences (Table S3). This contrasts sharply with the benefits of on-the-land cultural activities:

“*This land where I come from is very, very important…probably the only First Nation in northern Ontario or in Ontario that doesn’t run social assistance or welfare programs for our membership because the land looks after us. We have an abundance of fish, wildlife, waterfowl and stuff, and as a result, the land is our social welfare system, and we would like to keep it that way. We’ve got good, clean water and we can dip our cups into any of our river and creek systems without worrying.*”

(Chief George Hunter of Weenusk First Nation) [71] (p. 956)

##### **Meaningful Involvement in Decision-Making and Free, Prior, and Informed Consent** 

Meaningful consultation and participation in the decision-making process—with respect to development (or prior to development or no development scenarios) on First Nations homelands—was consistently brought up by First Nations’ leadership with regards to the planning of protected provincial parks and prior to the introduction of government bills that impact constitutionally protected Indigenous inherent rights and treaty rights (Table S4). For instance, Grand Chief Stan Louttit of Mushkegowuk Tribal Council [72] (p. 985) described his dismay:

“*The Premier [of Ontario] did not come to us and ask for our opinion in terms of the protected areas. There was an announcement one day that there would be protected areas of 250,000 square kilometers in our territory, much to our chagrin. We were quite shocked… [the Ontario] Ministry of Aboriginal Affairs, they’ve gone on the record as wanting to work with us, and then making arbitrary decisions like that without talking to us was very, very shocking.*”

Furthermore, the timing and location of consultation meetings were questioned:

“*[With respect to] legal duties to consult with First Nations… [First Nations] should be consulted without artificial timelines…First Nations and their members hold Aboriginal and treaty rights. They must be consulted directly…in our communities…when we talk about consultation…when we talk about NAN territory, we’re talking about 55 million hectares…there are three distinct groups within that territory [Crees, Oji-Crees, and Ojibwas]…there’s a legal requirement of the crown’s responsibility to consult with them, we would expect that an attempt be made to talk to those people in their own language so that they understand what is being proposed to them.*”

(Grand Chief Stan Beardy of Nishnawbe Aski Nation) [68] (pp. 828–831)

Moreover, the façade of power-sharing was exposed in the wording of statutes, whereby the Government of Ontario has the legal authority to override a First Nation government-approved land use plan—that protected land of significant cultural importance—if in the interests of the Government of Ontario on behalf of all other Ontarians:

“*[T]he government has the ultimate power, with the explicit discretion under the bill* [73]*, to override any land use plan and permits a new mine to be developed if it is in the economic and social interest of the province to do so. I take this as an insult. This is essentially saying that my community could prepare a land use plan, identify an area of land which, for whatever reason—whether cultural, traditional or environmental—is off limits for mining development and the government has the authority to basically say that there are economic and social interests of the province which are more important than my community’s interest and proceed to permit the mine.*”

(Chief Theresa Hall of Attawapiskat First Nation) [69] (p. 982)

This type of discretionary decision-making power on the part of the Government of Ontario spurred First Nations leadership to request free, prior, and informed consent (FPIC) with respect to both Bill 173 and 191 to protect their rights (Table S4). Chief Randy Kapashesit of the MoCreebec Council of the Cree Nation [74] (p. 957) specifically addressed this issue at one of the hearings:

“*There is something called free, prior, and informed consent. That’s been a big issue for Indigenous people the world over. In the process and in the context of this particular initiative, I see no evidence that that has in fact been achieved or even attempted to be achieved. Again, I remind you that the human rights of Indigenous people are being violated here as we sit before you. You can say that you’re not really doing that…but your actions…speak [otherwise]…In the course of developing…the Far North Act, I’ve seen no initiative to actually be engaged with our communities, to say, ‘What is it that you’re interested in?’ I see this more as an imposition, a continuation of a higher power at work, if you will, telling us that this is the way it has to be. ‘Never mind your human rights, never mind your historical rights; we’re not interested in that’: That’s what you’re saying by producing this kind of document and expecting us to participate, meaning that you haven’t actually spent any time to even develop an approach that achieves free, prior and informed consent.*”

#### 3.3.3. Indigenous Perspectives from across Canada

In their submission to the committee, Fort McKay First Nation [75] (p. 7) reported that their community members indicated that

“*Culture includes physical cultural sites, cultural practices, cultural landscapes, cultural values, and well-being… [and] that core cultural elements have been and will continue to be negatively impacted by industrial development. Members believe cultural impacts will lead to unacceptable change in the community.*”

##### **Land and Water, Sustainability, and Inherent Obligations** 

Similarly, the Manitoba Metis Federation [76] (p. 6) described “*their cultural identity and connection to the land*”, while Fort McKay First Nation [75] (p. 1) reported that the Fort McKay people “*used these lands for millennia; lands that are rich in the cultural heritage.*” In addition, when the Indigenous groups referred to the land, this reference was typically made in the context of their entire traditional territory or Indigenous homelands, as defined by them [77,78], not just reserve lands. This was why Peguis First Nation [79] (p. 3) stated that “*proximity is a myth*” in reference to culturally important sites—such as petroglyphs in the Whitehead region of Manitoba—because these sites have been and will always be sacred to the Anishinabe people of Manitoba, not just one First Nation. Furthermore, biophysical indicators alone cannot be used as proxies of impacts on Indigenous peoples’ rights because, from Indigenous perspectives, there are also interrelated cultural and spiritual aspects of importance; not accounting for this fact would lead to a ‘false equivalency’ [77,78,80]. Likewise, certain development project impacts on Indigenous rights cannot be mitigated by exercising the right elsewhere in ‘alternative areas’ [77,78].

For the Indigenous peoples of Canada, water has importance beyond travel, and the protection of water is of utmost importance [78,79,81,82,83,84,85,86,87,88]. As ‘custodians’ [89] or ‘keepers of the waterways’ [83], First Nations from across Canada “have repeatedly emphasized the cultural, economic and environmental importance of protecting all navigable waters” [90] (p. 11). The protection of all waterways is “*the only way to preserve, protect, and respect inherent and Treaty rights*” [91] (p. 7). Unfortunately, Bill C-69 ignored:

“*From an Indigenous perspective, the ability to travel by water to access fishing areas is inextricably linked to the health of those waters. Activities that impact navigation and the ability to fish have cascading effects that reverberate through a community: impacting the spirit of the water; the ability of the water to support aquatic and terrestrial species, including plants that are harvested or used in traditional activities; travel through First Nations’ territories; the ability to pass along cultural and ecological knowledge accumulated over generations; and undermining trading and family relationships among First Nations…and otherwise use water.*”

(First Nations Fisheries Council) [92] (pp. 2–5)

Other Indigenous groups specifically declared the importance of connections to land and water in relation to their identity and wellbeing (Table S5). Clearly, as stated by The First Nations Major Projects Coalition [93] (p. 3), “*A renewed relationship between the Crown and Indigenous people is inextricably tied to the environment.*”

Thus, it is not surprising that the Nunatsiavut Government [64] (p. 7) declared the “*sustainability of the environment and ecosystems are vital to Indigenous peoples.*” Adding further, the Algonquins of Ontario [94] (p. 3) recounted that

“*Our survival on this land for thousands of years required us to apply our teachings to ensure the protection of the lands and waters that we rely on. These teachings serve as the original instructions or ‘natural laws’ that were built into our way of life. ‘Sustainability’ is a modern term, but sustainability was long in practice by our people and our ancestors…We had, and continue to have, deep connections to the land. Protection and interaction with the lands and waters of our territory has been central to our existence for thousands of years. We have maintained this connection to the land despite the arrival of Europeans to our territory. Nonetheless this arrival has dramatically impacted our way of life.”*

In a like manner, Chief Maureen Thomas of Tsleil-Waututh Nation [95] (p. 13), in her presentation, communicated that

“*There are so many things that impact our well-being …jurisdiction…It’s inherent…to be stewards of our land. We’re here to protect it. We’re here to ensure that it’s there for our grandchildren down the road. There is nothing that is going to stop us from protecting it…When things come into our territory, we have to ensure that what is brought there doesn’t leave a lifelong risk that is going to extinguish our being on that territory for my children and grandchildren down the road.*”

Lastly, others [84,96] wanted the inherent jurisdiction of Indigenous peoples to be recognized in relation to environmental impact assessments and any type of development in their territories.

##### **Being on the Land, and Indigenous Languages and Knowledge Systems** 

Having a relatively intact environment is of paramount importance for the Indigenous peoples of Canada in the maintenance of their cultures [81] because a relatively intact environment allows for the participation of Indigenous peoples in on-the-land cultural activities (Table S5). Intact land, Indigenous languages, and Indigenous knowledge systems provide a cultural framework. In the words of the Assembly of First Nations Quebec-Labrador [90] (p. 10): “*Indigenous knowledge, traditional and contemporary, is at the heart of our identity and culture and must therefore be protected.*” Some did not agree with the term ‘traditional’; they believed that the use of the term Indigenous knowledge better captures “*the scope of Indigenous information*” [96] (p. 8). It was also emphasized that similar to Indigenous cultures, Indigenous knowledge systems were not (and are not) ‘frozen in time’ [78,80,81]. The term ‘traditional’ is a misnomer [82,97] because “*the use of the term…could exclude the evolution of Indigenous Knowledge that occurs over time in response to new circumstances and changes in the environment*” [98] (p. 16). Similarly, some contended that it was “*necessary to see Aboriginal and Treaty rights as dynamic geographically, culturally and temporally*” [77] (p. 7) to allow for “*future use of lands and waters for socio-economic and livelihood purposes*” [94] (p. 8). In particular, Bill Namagoose, Executive Director of the Grand Council of the Crees (Eeyou Istchee) [99] (p. 3), highlighted the point: “*We occupy and intensively use the entire area of Eeyou Istchee, both for our traditional way of [life hunting and trapping]…and increasingly, for a wide range of modern economic activities.*” Likewise, Wolastoqey Nation in New Brunswick [82](p. 6) presented that it was important to

“*Consider all uses of lands and resources, not just for traditional purposes… Strictly considering the current use of lands and resources for traditional purposes will have the effect of freezing rights in time, which would exclude the evolution of rights that occurs over time in response to new circumstances and changes in the environment.*”

##### **Sustainable Development** 

As asserted by the Nunatsiavut Government [64] (p. 11), “*Indigenous peoples have a tradition of sustainable, respectful development and use of the land.*” Nevertheless, this stewardship responsibility does not mean that Indigenous groups are all opposed to non-Indigenous development of their homelands [100], as long as it is sustainable development from Indigenous perspectives [90] (Table S5). Specific examples of this viewpoint include

“*While we are not opposed to all forms of development, we do believe that our collective Treaty obliges the Crown…to take steps to ensure that all developments are sustainable and to ensure that there enough lands of sufficient quantity and quality to sustain our rights, way of life, culture, livelihood and to ensure the health and safety of our people and our friends and neighbours of the Peace River country.*”

[101] (p. 1)

“*Nunavik Inuit are not opposed to development…large-scale development projects can represent significant economic potential for our regions and our communities…[but] there is an expectation within our communities that development projects will not be allowed to proceed unless every precaution has been taken to ensure that they are compatible with our understanding and respect for the environment, and that they uphold the maintenance of Inuit livelihoods, traditional practices, and the cultural identity.*”

[88] (p. 5)

From this point of view, important concepts, such as ‘ecological thresholds’ [102], ‘environmental and cultural thresholds’ [75], and ‘carrying capacity’ [103], must be considered in the context of sustainability and regional and strategic assessments [64]. Regional and strategic assessments would allow for the consideration of cumulative effects [95,96,104], which is of importance because cumulative effects were described by many Indigenous groups as a major threat to Indigenous peoples and their homelands [63,66,90,97,102,105]. In the words of Fort McKay First Nation [75] (p. 6), “*cumulative impacts have dramatic consequences to the environment, culture, social structure, health, traditional economies and Rights of Indigenous peoples.*” Indigenous groups discussed cumulative effects using a variety of phrases, such as ‘death by a thousand cuts’ [77,78,80,81,102,106], cumulative load [107], and cumulative impacts [63,64,98,103,108]. Regional and strategic assessments were seen as having the potential to permit sustainable development [64] by accounting for incremental impacts of urbanization and industrialization [104] on the environment and concomitant effects on Indigenous cultural wellbeing.

When development is of the non-sustainable type, Indigenous peoples lose the “*opportunity to continue to practice their traditional pursuits on the land. In some instances, the land or sources of water are destroyed and not available to provide sustenance to the local FN [First Nations] peoples after the project is complete and long gone*” [79] (p. 4). The detrimental impacts of development on the landscape, watershed, and airshed were mentioned in many of the submissions [75,77,83,102]. Several detailed examples are presented henceforth:

“*Our traditional territory has been heavily impacted by hydro dams and the rapid expansion of oil sands activities, including open pit and in situ projects… industrial activities…impact our rights and [need] to provide protections for our lands and waters. We rely on navigation protections to access our hunting, trapping and cultural areas.*”

[63] (p. 1)

“*[T]here has been significant industrial development…including open pit and in-situ oil sands mining, uranium mining, sand and gravel mining, forestry, and pulp and paper mills…provincial and federal environmental assessment and protection laws have failed…these activities have degraded the natural environment, reduced or extirpated numerous species of wildlife, brought sickness to our communities, and infringed upon our Treaty and Aboriginal rights…[our] territory is being destroyed, habitat fragmented, species are being lost, watersheds depleted, and water and air contaminated.*”

[102] (p. 1–4)

“*Air We Cannot Breathe’…we have signs up all over the place about sour gas…’Fish We Cannot Eat’…All of the fish in the reservoir system have high concentrations of methylmercury…’Land we Cannot Use to Hunt or Trap’…signs throughout the whole area that restrict our activity in those areas….’Animals We Cannot Eat’…caribou… eating contaminated soil…’Water We Cannot Drink’…signs throughout the territory about being careful not to drink the water…’Forests we Cannot Use To Camp’…signs are up that restrict us from camping through our areas…sloughing has been happening since they flooded.*”

[106] (p. 10)

Beyond the above-described impacts, the Native Women’s Association of Canada [65] (p. 8) has recognized that: “*Industrial projects in or near Indigenous communities can result in increased rates of violence against women…in the form of physical or sexual violence.*” Ultimately, as asserted by the Nunatsiavut Government [109] (p. 3) in their presentation:

“*This bill (Bill C-69) continues the practice of using the power of [colonial] laws to license the slow and steady genocide of Canada’s Indigenous peoples in the name of the public interest. We are asking you to stop that, here and now, in this bill.*”

##### **Meaningful Involvement in Decision-Making, and Free, Prior, and Informed Consent** 

As noted by the Nunatsiavut Government [64] (p. 8):

“*Indigenous peoples, our cultures, territories and rights, have been rendered less and less sustainable through the advancement of the economic and social interests of Canada as a whole…decision-makers do not exercise restraint or caution, nor do they take precautionary measures, when possible, harm to Indigenous peoples is weighed against perceived benefits that a project will bring to Canada generally.*”

This is why Indigenous peoples of Canada have vied for empowerment to look after their homelands and affairs in the colonial setting of Canada (Table S6). There must be “*significant recognition of Indigenous governance and Indigenous norms and laws*” [96] (p. 13), with Indigenous perspectives being given equal weight compared to the non-Indigenous (or western) perspective in decision-making processes [78,81]. The definition of Indigenous governing bodies cannot be made from a colonial perspective [77,78], “*Indigenous groups must be recognized as jurisdictions as a starting-point.*” [95] (p. 4) In essence, Indigenous governments must be respected and treated the same as other levels of government [85,90,91,108] because, as forcibly stated by the Assembly of First Nations [98] (p. 9):

“*First Nations are rights holders, who hold inherent and constitutionally protected rights set out in their own governance and legal systems, as well as under Section 35 of the Constitution. In practice, this means that First Nations rights cannot be undermined by colonial interpretation of their rights (i.e., s.35). Instead, First Nations must first interpret and describe their inherent rights, grounded in Indigenous law, Indigenous legal traditions, and customary law. These legal orders, which lay the foundation for First Nations’ concepts of self-determination and sovereignty, are essential to starting true “Nation-to-Nation” dialogues and expressing the respect for our rights and title. For the millennia, prior to contact with European explorers, First Nations exercised control over their territories through their own governance authorities.*”

Indigenous Nations of Canada must be recognized and empowered as ‘joint decision-makers’ [104] who have shared stewardship of their lands and waters with the Government of Canada [85]. This federal responsibility should not be downloaded to the provinces, such as the Government of Quebec, which are known to utilize a lower standard of consultation with Indigenous peoples compared to the federal government [83,89,103]. The Government of Alberta has also been shown to be unwise stewards of Indigenous lands [101,110].

Of particular concern to Indigenous Nations who had previously negotiated and signed comprehensive land claim agreements (or modern treaties) with the Government of Canada was that their negotiated agreements were not mentioned specifically in Bill C-69. This was disconcerting to the Indigenous Nations because these agreements already had multi-jurisdictional decision-making protocols in place with respect to environmental assessments, and these agreements were constitutionally protected [88,109,111,112,113].

The importance of free, prior, and informed consent in development project decision-making was highlighted in the submissions: the need for “*obtaining the free, prior, and informed consent of Indigenous peoples before proceeding with economic development projects.*” [65] (p. 2); “*the legislation should make specific references to…the principal of free, prior and informed consent*” [93] (p. 2); should be a “*requirement to obtain Indigenous groups’ free prior and informed consent*” [77] (p. 6); and “*explicit mention should be made…to the requirement to obtain Indigenous peoples’ free prior and informed consent*” [78] (p. 5). Nonetheless, for free, prior, and informed consent, “*there isn’t a one-size-fits-all. There needs to be dialogue among governments and Indigenous peoples to establish how free, prior, and informed consent will be obtained and respected.*” [114] (p. 21). Further, the incorporation of free, prior, and informed consent into the decision-making process may “*advance reconciliation in Canada*” [102] (p. 2).

However, for true reconciliation to occur, the Government of Canada cannot unilaterally interpret the meaning of reconciliation; Indigenous perspectives are of utmost importance:

“*Government is defining what reconciliation relations are as a priori to extinguishment of rights and title under a planned federal *“*legislative framework*”* to transition bands currently under the Indian Act into *“*self-government*”* agreements, or Comprehensive Claims Agreements/*“*Modern Treaties*”*, which the government regards as *“*self-determination*”*…First Nations rights and title cannot be undermined by colonial interpretation of reconciliation.*”

[83] (p. 5)

Lastly, Chief Jim Boucher of Fort McKay First Nation [110] (p. 18) made the observation that “*It has been easy for governments to talk about reconciliation, but more difficult to translate those words into action. I have yet to see a true example of reconciliation from this government.*”

### 3.4. A Brief Overview of the Results in Relation to Study Objectives

#### 3.4.1. To Conceptualize Wellbeing from a Fort Albany First Nations Elders’ Perspective and Identify Valued Aspects of Wellbeing

Fort Albany First Nation Elders conceptualized wellbeing in a number of ways related to physical and mental health, balance and harmonious relationships, relatedness, honouring inherent obligations, traditional lands, and going out on the land. Their concept of wellbeing was scaled; wellbeing was described not only at the level of the individual but also at the family, community, and nation levels. Fort Albany First Nation Elders identified the environment and, in particular, the Cree traditional homeland as being of utmost importance for cultural wellbeing. The land and its resources (e.g., traditional foods) were described as gifts from the Creator. The land allowed for important on-the-land activities (e.g., spiritual pursuits, the harvesting of traditional foods), and with these gifts, there were obligations (e.g., maintaining the harmonious balance and interrelationships between everything) that needed to be met. The land was essential for Cree cultural wellbeing as it was the basis for the Cree language, Cree knowledge, Cree spirituality, cultural traditions, and the forum for sharing Cree knowledge—in other words, Cree identity.

#### 3.4.2. To Explore What Is Important for Wellbeing from a Northern Ontario First Nations’ Perspective, and to Examine at a National-Level, What Is Important for Wellbeing from First Nations, Inuit, and Métis Perspectives

Although no definition of wellbeing was apparent for northern Ontario First Nations and Indigenous leadership and organizations from across Canada in the data examined, several types of wellbeing were mentioned (e.g., economic, political, social, and cultural). This holistic and multidimensional conceptualization of wellbeing was collectivist in perspective, with the land being the connecting thread binding together the different types of wellbeing. Of importance, wellbeing was predominantly discussed at the cultural level by regional and national Indigenous leadership and organizations. Even in acknowledging the cultural heterogeneity with respect to Canadian Indigenous nations, four concordant themes were identified regionally in Ontario and nationally, with respect to what was important for cultural wellbeing: land and water, sustainability, and inherent obligations; being on the land, and Indigenous languages and knowledge systems; sustainable development; and meaningful involvement in decision-making, and free, prior, and informed consent.

## 4. Discussion

### 4.1. What Is Wellbeing?

To summarize from a Fort Albany First Nation Elders’ perspective, wellbeing was described in terms of physical wellbeing (e.g., health), mental wellbeing (e.g., Indigenous identity, happiness), spiritual wellbeing (e.g., balance of the four components, such as body, mind, spirit, and emotions; and harmonious relationships), social wellbeing (e.g., family), and cultural wellbeing (e.g., ‘going out’ on the land). Interestingly, land was the connecting thread between all types of wellbeing and was described as a place to practice cultural traditions, pass on Indigenous knowledge and language, and improve one’s wellbeing. Wellbeing was also described at different scales from the individual, to the community, to the nation, to the world. Different types of wellbeing were not mentioned from a northern Ontarian First Nations perspective, but from Indigenous perspectives from across Canada, wellbeing was communicated as economic wellbeing, political wellbeing, social wellbeing, and cultural wellbeing.

In Canadian studies, wellbeing has oftentimes been placed in the context of health or vice versa. For instance, it has been reported that Métis women in Manitoba conceptualize health in the physical sense, while wellbeing was described in a more holistic manner that included spiritual (e.g., being supportive), emotional (e.g., practicing traditional activities, being a positive role model), mental (e.g., respecting the views of others), and physical (nutrition and physical activity) dimensions [115]. Collectivism was also reflective in the women’s conception of wellbeing rather than individualism, which dominates non-Indigenous views of wellbeing [115]. In another study with Elders in two Anishinaabe communities in Ontario, the Elders identified health holistically with social, cultural, environmental, emotional, spiritual, and physical dimensions being of importance to a person’s wellbeing [116]. For the Whapmagoostui Cree Nation of Quebec, wellbeing was described in the context of being on the land and connected to all things that make a person Cree or ‘living well’ (*miyupimaatisiiun*), such as taking part in cultural activities (e.g., hunting, gathering) and consuming wild meats and fish [117]. To the point, one Whapmagoostui Cree Elder queried: “*How, can we ‘be alive well’ if the land is not?*” [117] (p. 13). This same sentiment was expressed by the Quebec Cree regional Nishiiyuu Council of Elders [118] (p. 1): “*What is done to our land is done to our people.*” Cree peoples’ health and wellbeing are linked to the state of the land [119]. For the Opaskwayak Cree Nation of Manitoba, the ‘good life’ (*mino-pimatisiwin* in their dialect) refers to an on-going process of living a balanced-harmonious life through interconnections with the physical, emotional, spiritual, and mental aspects of their life [120]. A ‘good life’ in Dene is *honso aynai*, and in Dakota, *tokatakiya wichoni washte* [120]. With regards to the Anishinaabe, the concept of a ‘good life’ (or ‘living well’) is referred to as *mino-bimaatisiwin* [121], and to achieve a good life, life must be lived holistically by balancing body, mind, feelings, and spirit [122], with many paths to achieving it [120,121]. With reference to living a good life, it is a process and a goal strived towards, individually and collectively [121,123]. Thus, the wellbeing concept is scalable from individuals, to families, to communities [124], to nations [11] and to the universe [125,126] with land being of paramount importance.

Likewise, the concept of *buen vivir* (‘Good Life’) in Ecuador comes from Andean Indigenous tradition [127] and has five pillars: “*harmony with Nature, respect for the values and principles of indigenous peoples, satisfaction of basic needs, social justice and equality as responsibilities of the state, and democracy*” [128] (p. 20). Further, the Matsigenka of the Peruvian Amazon believe in the concept of *shinetagantsi* (happiness or becoming happy) with the premise of a good life being collectively associated with productivity and the maintenance of harmonious relationships (physical, social, and spiritual) with the environment [129]. In New Zealand, *hauora* for the Māori is a holistic view of health and wellbeing comprised of physical wellbeing, mental and emotional wellbeing, social wellbeing, and spiritual wellbeing, with their land-based culture being an overarching umbrella [130]. For northern Indigenous peoples of the Russian Federation Republic, wellbeing was found to be closely associated with traditional lands and practices [24]. Further, Alaska Natives of the United States of America used the phrase ‘keeping busy’ to describe health and wellbeing, in reference to being on the land, consuming traditional food while also respecting Elders and nature [131]. In Australia, it is generally recognized that ‘country’ (i.e., land, water, and air) is central to Indigenous peoples’ wellbeing [132]. Indeed, Indigenous peoples worldwide know that the health and wellbeing of the people mirror the state of the environment [133]. Clearly, land and culture are of utmost importance to Indigenous peoples’ wellbeing around the world. It also should be emphasized that when the term ‘land’ is used in reference to Indigenous peoples, land is shorthand for land aboveground and belowground—water, sometimes mentioned with land to emphasis its cultural importance—and air [134]. Land is also used interchangeably with ‘Mother Earth’ (Bell 2013), and ‘land-based’ encompasses water and air [135].

### 4.2. What Is Cultural Wellbeing?

In brief, Fort Albany First Nation Elders conceptualized cultural wellbeing in relation to the land, traditional procurement activities (e.g., hunting, fishing, and gathering) and traditional foods, continuation of the Cree language, and the practice of spirituality (or conversely not engaging in unhealthy activities such as alcohol consumption or drugs) reconnecting with their ancestors/spirits and rejuvenating oneself. Being on the land was described as regaining control of one’s environment with the chance to exercise their inherent duties and establish (or re-establish) harmonious relationships with the environment and culture. Importantly, cultural wellbeing was conceptualized in the context of land, specifically Cree ‘traditional land’ or the *Omushkego* Cree homeland, that is, at the nation level. First Nations people of Canada think about land (and culture) in the context of their traditional homelands because treaties were signed for the sharing of their traditional homelands but not their ‘reserve’ lands—between First Nations’ leaders and the British Crown (on behalf of Canada)—at the scale of nation-to-nation [28,29,136].

Culture has been defined in the Preamble to the UNESCO [137] (p. 18) Universal Declaration on Cultural Diversity as “*the set of distinctive spiritual, material, intellectual and emotional features of a society or social group, and…encompasses, in addition to art and literature, lifestyles, ways of living together, value systems, traditions and beliefs.*” However, there is ambiguity associated with how cultural wellbeing has been defined and/or measured [138]. Perhaps the two core themes with respect to Indigenous wellbeing identified by the UN Permanent Forum on Indigenous Issues [24]—that is, identity, land and ways of living (subthemes: languages and Indigenous knowledge, cultural activities, sovereignty of lands, health of communities and ecosystems); and Indigenous rights to, and perspectives on, development (subthemes: Indigenous governance, free, prior, and informed consent, participation in matters affecting indigenous peoples’ wellbeing, and self-determination)—should serve as a starting point in this discussion. Indeed these two major themes bear resemblance to what was identified in the present study from the perspectives of northern Ontarian First Nations (Tables S3 and S4), and Indigenous leadership and organizations from across Canada (Tables S5 and S6), with respect to what is important for cultural wellbeing (or the valued components of cultural wellbeing)—that is, land and water, sustaining the environment, and honouring inherent obligations; being on the land, and indigenous languages and knowledge systems; and meaningful participation in the decision-making process, and free, prior, and informed consent.

In Canada, it has been reported that 63% of First Nations respondents to a health poll thought that loss of land and culture impacted negatively on their health (and wellbeing) [139]. Meanwhile, in another survey, First Nations respondents identified the revival of Indigenous cultures and traditions (e.g., Indigenous languages, healing practices)—with the caveat that First Nations’ traditional practices vary greatly across Canada—as potential ways towards the improvement of health (and wellbeing) [139]. It has been suggested that culture in itself can be considered an intervention moving toward wellness [140]. Unsurprisingly, northern Indigenous researchers speak of their responsibility “*to keep the land alive,*” as this is a necessary condition for the survival of their peoples [141] (p. 101). Somewhat perplexing in the academic literature, the concept of wellbeing is often only applied at the individual, family and community level [142], even though culture is often identified as a central feature of wellbeing [11].

Correspondently, globally, strong connections with one’s Indigenous culture have been positively associated with an individual’s health and wellbeing [143,144] and/or reduced risk-taking behaviours [145]. Furthermore, Indigenous individuals typically think not only about themselves but also about the wellbeing of the whole community as they have a more relational view of health and wellbeing [143]. It is difficult to disentangle wellbeing for Australian Indigenous peoples at an individual level from wellbeing at familial and community levels [18]. For Native Hawaiians, health is viewed holistically and more than physical and mental wellbeing; health includes the concepts of righteousness and balance between relationships with other people, the spiritual world, and the land (*aina*) [146]. In a qualitative study of Native Hawaiian adults, major themes were identified that included *aina* is everything and *aina* is an indicator of health and wellbeing in individuals, families, and communities [146]; these sentiments mirrored results in the present study and what has been published in the literature. Lastly, as noted by the United Nations Permanent Forum on Indigenous Issues [147] (p. 2): “*Land is the foundation of the lives and cultures of indigenous peoples all over the world.*”

### 4.3. What Is Important for Cultural Wellbeing?

It should be stressed that the identified themes or the valued components of cultural wellbeing (Tables S3–S6) from Canadian Indigenous perspectives have been artificially generated and are not mutually exclusive. In reality, one theme—land—could have been used. All the other themes were linked either directly and/or indirectly to land, but the use of several themes allowed for a more linear discussion. Equivalently, Indigenous Community Health Representatives from across Canada articulated that land has been and continues to be foundational to Indigenous cultures, being central to their societies [148]. In a like manner, Dene First Nation youth from the Northwest Territories identified a connection to the land as an important determinant of health and wellbeing for them [149]. Recently, Indigenous cultures and homelands have been conceptualized as one holistic determinant of health and wellbeing [150].

Historically in Canada, conflicts over land between non-Indigenous Canadians and Canadian Indigenous peoples have occurred due to the fact that land, from colonial perspectives, was viewed as an uninhabited hinterland, a commodity to be exploited for capitalistic gain rather than a relative to be respected and safeguarded [151,152,153]. Policy makers and planners must see the land as more than physical space, for without this understanding, modern-day colonialism will continue, and tension between non-Indigenous and Indigenous populations will be perpetuated [136,154]. As noted in the Ipperwash Inquiry mandated by the Government of Ontario after the death of Dudley George at Ipperwash Provincial Park—the fundamental conflict between settlers and First Peoples is typically about land [136]. The Indigenous legal scholar Borrows [155] (p. 3) succinctly states: “*Aboriginal and non-Aboriginal peoples conflict over lands and resources [is] because of the lack of recognition and affirmation of Aboriginal rights and perspectives… Aboriginal peoples regard their traditional lands as sacred; it is integral to their culture and identity… [and their homelands are needed to] preserve their ancient relationships.*”

#### 4.3.1. Land and Water, Sustaining the Environment, and Honouring Inherent Obligations

In summary, Fort Albany First Nations’ Elders identified Cree “*traditional land*” as being a gift from the Creator, being of utmost importance to cultural wellbeing. The land provided resources and allowed for traditional cultural activities, such as spiritual and physical sustenance pursuits, and the forum to meet their stewardship obligations to use the land wisely, maintaining the harmonious balance and interrelationships between everything. Of importance to cultural wellbeing—and related to the land and inherent obligations—were the Cree language, Cree knowledge system, Cree cultural traditions, and the horizontal and intergenerational sharing of knowledge. Lastly, Cree culture was described as dynamic and changing with the world.

Likewise, the northern Ontarian First Nations’ leadership mentioned how the land (and water) provided sustenance to them. In addition, the land (and water) was described as being foundational to their identity and wellbeing, including cultural wellbeing. In brief, First Nations’ leaders espoused that the land was not only their ancestral home gifted to them by the Creator; the land was part of who they were as a people because of their intimate connection to the land and everything in creation (Table S3). However, there were inherent obligations that came with this gift of land. These inherent obligations or rights were bestowed by the Creator; thus, they could not be extinguished by colonial governments (Table S3). Inherent obligations included stewardship of the land, including the sustainable use of the land.

Similar sentiments to the ones presented by northern Ontarian First Nations’ leadership were voiced by Canadian Indigenous groups from across Canada through their leadership and/or organizations. Cultural identities and heritages were declared to be connected to the land and water (Table S5). Since Indigenous cultures were said to include physical, cultural sites, cultural landscapes and waterscapes, cultural values, cultural practices, Indigenous languages, and Indigenous knowledge systems—it is not surprising why land and water (and air)—were regarded as paramount for cultural continuity and wellbeing. Foundational to Indigenous peoples’ ways of life (or cultures and wellbeing) was the honouring of their inherent obligations or ‘natural laws’ that included the concept of stewardship of the environment and sustainability for future generations. This was especially poignant, noting that the Indigenous Nations of Canada hold constitutionally protected inherent and treaty rights, as they stated. It was emphasized by Canadian Indigenous peoples that stewardship of the land was not just reserve lands but their Indigenous homelands. Likewise, Indigenous peoples were the ‘custodians’ or ‘keepers’ of the waterways. For the Indigenous peoples of Canada, waterscapes were professed as being important for sustenance, travel, and culture. The protection of Indigenous homelands and all waterways was seen as the only way to ultimately protect Indigenous inherent and Treaty rights, in essence, their cultural wellbeing. Indigenous peoples’ connections to land and water were identified as being of great importance to their identity and wellbeing (Table S5).

In studies from across Canada, Indigenous peoples have described their interconnection with the land in a variety of ways. For example, being a part of the land (e.g., the Blackfoot People of Alberta [156]), sprouting from the land (Mikmaq of Nova Scotia [157]), people of the land (Cree of Ontario [158]) or they are the land (Anishnaabe of Ontario [116]), and being complete with the land (Inuit of Nunatsiavut, Labrador [159]). Additionally, Anishinaabe Elders report that “*the land is everything*” [116] (p. 30), and many First Nation Elders hold the belief that “*everything is related*” [122] (p. 386), while the Innu of Labrador refers to the land (*Nutshimit*) as being core to their wellbeing [154]. The land was (and is) where Indigenous peoples stayed busy and connected to their culture [160], partaking in social (e.g., ceremonies, feasting, sharing) and traditional (e.g., hunting, fishing, gathering) activities honouring their inherent obligations (e.g., principles of reciprocity, sustaining the land for the present and future generations) [154,158]. The land provides sustenance (e.g., game meats, fish, berries, etc.) [161,162,163,164], but Canadian Indigenous peoples are impacted disproportionately by food insecurity [165,166,167]. Thus, food sharing is a socially and culturally important activity in Canada [161,166], as well as worldwide in Indigenous communities [168], and food sharing networks have been shown to be extensive within and between communities [169,170] and important to individual and cultural wellbeing [170].

Understandably, the wellbeing of Indigenous peoples has been directly tied to sustaining the environment [171]. In order to sustain the environment and their ways of life, Indigenous peoples have followed their ‘original instructions’ (e.g., balance and harmonious relationships) [123,157,161]. McGregor [121,172,173], an Anishinaabek scholar, discussed these inherent ‘legal’ obligations in the context of considering all relationships in the cosmos, including the spirit world. Meanwhile, Bell [126] described the original instructions of the Anishinaabe as the Seven Original (Ancestral) Teachings (e.g., harmony and wellbeing; inherent autonomy; interdependence and interrelationship of all things; respect). The Mi’kmaw refer to relational obligations [157], and the Cree have reported similar sentiments in that it is their inherent duty and right to sustain the environment for future generations [158]. In Canada, Aboriginal rights and treaty rights are constitutionally protected in the realm of common law, but these Aboriginal (or inherent rights) have existed for millennia prior to the existence of common law [28]. Of note, there is an ongoing movement toward the revitalization of Indigenous laws, especially in the context of protecting the environment [28].

Around the globe, Indigenous peoples have also asserted that their cultures do not need state recognition of these Indigenous rights to validate their existence [174]. Importantly, Indigenous peoples’ inherent governance over their homelands for millennia has received recent attention as positive models for the settler states in the context of environmental sustainability internationally [174,175,176]. Interestingly, concordant values of relatedness (e.g., no distinction between the animate and inanimate, and inter-relatedness), respect (e.g., respectful relationships and not wasting gifts, taking only what was needed), and reciprocity (e.g., honouring inherent responsibilities such as stewardship) have been identified for Indigenous peoples on opposite sides of the Pacific Ocean, that is, the Māori of New Zealand, and First Nations of the west coast of Canada [177]. Further, Panelli and Tipa [178] described how from Māori perspectives, Māori culture-environment relations and customary obligations support individual, family, and collective wellbeing by providing the foundation of individual and cultural identity in the context of tribal territory [178]. Place is a concept that has human context but also cultural commitments where obligations are met [152]. In the United States of America, the Lakota of South Dakota are also connected to places or their cultural homelands [179]. Their cultural homelands are more than physical landscapes, their homelands are cultural landscapes that imbue identity and interconnectedness with the land through Lakota language, ceremony, spirituality, and history [179]. Cultural landscapes have been described by UNESCO [137] (pp. 15–22) as “*the ‘combined works of nature and of man’… [and] often reflect specific techniques of sustainable land-use, considering the characteristics and limits of the natural environment they are established in, and a specific spiritual relation to nature.*”

#### 4.3.2. Being on the Land, and Indigenous Languages and Knowledge Systems

Of importance to cultural wellbeing and related to the land and inherent obligations were the following, as identified by Fort Albany First Nations’ Elders: Cree language, Cree knowledge, Cree cultural traditions, and the horizontal and intergenerational sharing of knowledge. Lastly, Cree culture was described as dynamic, changing with the world.

Similarly, First Nations’ leadership in northern Ontario described the land as being important to cultural wellbeing, as the land, Indigenous languages, oral history, and knowledge systems were inseparable; being on the land allowed for cultural stories, history, laws, customs, and languages to be shared and perpetuated (Table S3). From a First Nations perspective, typical non-Indigenous derived measures of wellbeing (e.g., economic wealth and housing) were viewed as of secondary importance compared to land, language, and culture. In a like manner, Indigenous leaders and organizations from across Canada mentioned that a relatively intact environment was important for the participation of Indigenous peoples in on-the-land cultural activities (Table S5), including the transmission of Indigenous languages and knowledge systems. It was emphasized by Indigenous groups from across Canada that Indigenous knowledge systems and Indigenous cultures are dynamic and constantly evolving in response to a changing environment.

For Labrador Innu, being on the land allows them the “*freedom to be Innu*” to learn and share Innu knowledge and partake in cultural activities to strengthen relationships with humans and non-humans and perpetuate Innu identity [154] (p. 7). In the same way, Labrador Inuit being on the land is central to their identity, as it allows for hunting, fishing, trapping, and other cultural activities that strengthen social relationships and linkages to the land being a source of wellbeing [159]. When queried about happiness, Inuit in Nunavut mentioned spending time with family talking and sharing food, and being on the land most often [180]. For Ontario Cree, partaking in cultural activities (e.g., hunting, fishing, trapping) provides not only mental and spiritual wellbeing benefits but also physical wellbeing benefits [181]. In point of fact, participation in the longest-running on-the-land program in the world—the Quebec Cree Income Security Program—was shown that participation in cultural on-the-land activities was associated with higher levels of vigorous and moderate physical activity, and higher concentrations of omega-3 polyunsaturated fatty acids in blood [182]. With respect to the Dene, the importance of being on the land is more than being culturally active, it has been reported as being physically active; in other words, “*cultural promotion is health promotion*” [183]. In an on-the-land beaver harvesting program, Cree participants identified through photovoice and semi-directed interviews other key elements of wellbeing in addition to physical activities, such as strengthening identity (cultural connection, cultural continuity), healing (time on the land, physical and emotional healing), knowledge sharing, and strengthening familial and social relationships [184]. Similarly, in a goose harvesting program, these wellbeing aspects were identified as being important: being on the land (e.g., enjoying the view, new experiences, familiarity of being back home); social networks (e.g., making new friends, seeing old friends); sharing activities (e.g., food and knowledge); and transfer of Indigenous knowledge (e.g., vertical transmission from Elders to youth; vertical transmission from Elders to adults; and horizontal transmission from adults to adults) [170].

As noted for the Mi’kmaw, cultural memory is imbued in the land [157], while the Anishinaabek ways of knowing and being are embedded in land and language to the extent where “*Language is land and land is language*” [151] (p. 1). Land has been professed to be the most fundamental part of Indigenous life, with languages arising out of the land [135], and land has been referred to as an important teacher [135]. There is significance in naming because in Nunavut, the land speaks in the language of the Inuit; that is, Inuktitut was born through human relationships with land and water [134]. For other Indigenous peoples, “*The voice of the land is in our language…We sprang from the land and the language (or languages) sprang from us.*” [185] (p. 18) For the Anishinaabeg(k) peoples and many other Indigenous groups, if you do not know the land and language, you cannot know its naming, its ceremony, its song, its stories, its history or, in essence, its voice [121,123,141,153,172,173]. Just as important, Indigenous languages are expressions of worldviews, not just words; for instance, in Anishinabemowin, most nouns are animate because most of the world is believed to be animate, that is, they have spirit [123]. It follows that when an Indigenous language ceases to exist, a philosophy and a way of thinking disappear [11,185]. This is why there is great concern about the decreasing activity on the land and the loss of Indigenous knowledge [161,186,187]. While Indigenous knowledge is contained within the land, this knowledge also exists within the people of the nation [126,188]. Thus, programs to revitalize and/or strengthen cultural wellbeing, including Indigenous knowledge systems across Canada, have been implemented. Some have been on the land [189,190], some in multiple settings [191], and others are at various stages of implementation [73].

In Australia, a national qualitative study identified five foundational components of wellbeing for Aboriginal and Torres Strait Islander adults, with three interconnecting aspects of family, community and culture [144]. The five components of wellbeing were identified as such: belonging and connection (e.g., the importance of family, community and culture, and maintaining connection to one’s Country was seen as being paramount to wellbeing); holistic health (i.e., multidimensional wellness); purpose and control in the context of stability (e.g., employment, familial and cultural responsibilities); dignity and respect (i.e., the way they are perceived and treated); and basic needs (e.g., housing, money) [144]. Overall, the concept of wellbeing was beyond the level of the individual and was collectivist-based [144]. In a systematic review of Indigenous peoples of Australia and their perspectives on wellbeing that did not include the above study, similar non-mutually exclusive domains were identified: autonomy, empowerment and recognition (e.g., agency, self-determination); family and community (e.g., cultural connectedness); culture, spirituality and identity (i.e., interrelated and multidirectional relationships); Country (i.e., a holistic concept of land, belonging to the land); basic needs (e.g., food, money, housing); work, roles and responsibilities; education; physical health; and mental health (i.e., social and emotional wellbeing) [192]. The findings of the present Canadian study, the aforementioned Australian studies [144,192], and others [145] confirm that Indigenous peoples in Canada and Australia conceptualize wellbeing multidimensionally with the centrality of land. In a like manner, a systematic review of wellbeing and Indigenous peoples in Canada, New Zealand, and the United States revealed that the concepts of health and wellbeing were commonly viewed by Indigenous peoples as holistic and collectivist [143]. Additionally, concordant aspects of wellbeing—identity, connection, balance, self-determination, and the importance of land—were noted across countries, although themes varied [143]. Nevertheless, there were areas where perspectives diverged, such as basic needs, where Indigenous peoples of Canada and the USA included adequate housing and food security, and where the Māori of New Zealand did not mention these aspects directly [143].

#### 4.3.3. Sustainable Development

In summary, the First Nations of northern Ontario emphasized that they were not against development, but development must be sustainable from a First Nations perspective (Table S3). If not, the land will be irreparably damaged—‘a land of disaster’—and First Nations’ cultures would be devastated. Many Indigenous organizations from across Canada held similar sentiments and identified cultural connections to places rather than spaces; this contrasted sharply with the non-Indigenous concept of space leading to ‘false equivalency’ and the assumption that inherent and Treaty rights could be exercised in ‘alternative areas.’ Accounting for these disparate viewpoints leads to a better understanding of why Indigenous peoples of Canada have advocated for respectful and sustainable development; Indigenous cultures are place-based, not space-based. This is why many Indigenous leaders and organizations across Canada have communicated that they are not against development per se, but development must be sustainable from Indigenous perspectives (Table S5) since a relatively intact environment is vital for the continuation of Indigenous peoples and their culture. Inherent obligations of Indigenous peoples to the environment manifest themselves in the terms they use for sustainability. For instance, ‘environmental and cultural thresholds,’ ‘carrying capacity,’ and ‘cumulative effects’ (or ‘cumulative impacts’). When the development was of the non-sustainable type, detrimental environmental impacts included but were not limited to flooding, habitat fragmentation, pollution of the landscape, watershed, and/or airshed, with Indigenous sustenance and cultural activities severely or irreparably impacted. Other stated detrimental impacts of development included violence against women which impacted not only their individual wellbeing, but also the cultural wellbeing of their respective Indigenous groups due to the important role that women play in the transfer of Indigenous knowledge.

In Canada, since land has commonly been mentioned as key to Indigenous wellbeing, the importance of the environment in the broadest sense and sustainable development to Indigenous wellbeing cannot be overstated [139,193]; the maintenance of the environment in a prosperous state is essential for Indigenous peoples’ wellbeing [139]. There must be the realization that honouring reciprocal relationships maintains balance and that balance creates harmony—from this perspective harming the land will inevitably harm the people. For example, environmental contamination and the concomitant contamination of traditional foods (e.g., game meats, fish) due to development across Canada [157,194,195,196,197] and around the world are well-documented [168]. Thus, preserving the land for the present and future generations is of utmost importance [118]. Nevertheless, there are Indigenous organizations that are not opposed to the wage earner economy and development as long, as development is sustainable and compatible with Indigenous worldviews [118,198].

Accounting for the fact that globally, Indigenous peoples are heterogenous, there still exists a commonality between the groups in the recognition that Indigenous stewardship practices represent one of the oldest forms of sustainable interactions with the environment [123,174,199]. Moreover, non-Indigenous societies have recently become more interested in Indigenous ‘management’ (i.e., stewardship or custodianship) of their homelands, in the context of making the world more sustainable with respect to biodiversity conversation and climate change, among other things [175,176]. Perhaps a values-led approach—that is, management where objectives are guided by values, in particular, those that connect people to place and form the basis for sustained relationships—could lead to more sustainable development in Indigenous homelands [177]. Another approach that has been suggested would be to designate places of high cultural significance and critical to Indigenous cultural identity and wellbeing, “*Cultural Keystone Places*” [200] (p. 427). Lastly, to protect and manage rivers around the world more in keeping with Indigenous worldviews—rivers in New Zealand, India and Colombia were granted the status of legal entities in 2017 [201]. Specifically, and most notably, the Whanganui River in New Zealand was in the context of the water-based guardianship responsibilities of Māori people [201,202,203].

#### 4.3.4. Meaningful Participation in the Decision-Making Process and Free, Prior, and Informed Consent

In order to protect the land and cultural wellbeing, northern Ontarian First Nations’ leadership consistently identified their meaningful involvement in development decision-making using the United Nations Declaration on the Rights of Indigenous Peoples principle of free, prior, and informed consent, in contrast to the existing unilateral decision-making power of the Government of Ontario (Table S5) [34]. In a like manner, Indigenous people from coast to coast in Canada articulated that empowerment to look after their homelands and affairs come through meaningful involvement in decision-making, and the obtaining of free, prior, and informed consent (Table S6) [204]. Involvement must be on a nation-to-nation basis with joint decision-making shared with the original occupants of Canada, and there must be recognition of Indigenous inherent rights with Indigenous perspectives granted equal weight to the non-indigenous perspective in the decision-making process. Only in this way could the Government of Canada obtain true reconciliation with the Indigenous peoples of Canada, from Indigenous perspectives from across Canada.

In Canada, meaningful participation in the decision-making process, especially in the context of the development of Indigenous homelands, has been viewed as one way to empower Indigenous peoples in the context of looking after their land and affairs and having a real say on development in their homelands. For example, Chief Andrew Solomon [193] (2012, p. 1) of Fort Albany First Nation forcibly espoused with respect to the Kabinakagami River Water Power Project that “*The participants at the community meeting…highlighted the relationship between the land, the river and our wellbeing over past, current and future generations. It is this connection that requires us to take an active role in this proposed development and ensure a proper EA [Environmental Impact Assessment] process is followed; one that includes our knowledge and influence.*” Although there exists the duty to consult and accommodate in Canada—based on Aboriginal inherent and treaty rights being entrenched in the repatriated *Canadian Constitution Act, 1982*, and domestic case law [205]—this duty to consult does not apply to the law-making process [49]. Thus, many development projects across Canada have been exempted from the Environmental Impact Assessment processes and the duty to consult Indigenous peoples through recent legislation [34,204]. This situation sharply contrasts the circumstance in Norway, whereby consultation and free, prior, and informed consent obligations are rooted in international law (e.g., the International Labour Organization’s Convention on Indigenous and Tribal Peoples (ILO C-169)) [205]. However, international law has been highlighted in the discourse pertaining to the implementation of the *United Nations Declaration on the Rights of Indigenous Peoples in Canadian law* [205], and with the ascent of Bill C-15 (*An Act respecting the United Nations Declaration on the Rights of Indigenous Peoples*) [206] on 21 June 2021, there should be more clarity in the future on the legal duty to consult obligation and the free, prior, and informed consent process in Canada [207].

In addition, the right to governance (e.g., self-government) has been identified as a determinant of wellbeing by the Assembly of First Nations [139], and the right to self-determination is considered an inherent right by Indigenous peoples [208]. Therefore, the Government of Canada must move away from its colonial-assimilative path in order to move towards reconciliation, and in the process, acknowledge the importance of multiple perspectives [209,210] and accept that no knowledge system is better than the other, just different [158]. By the same token, the land must be viewed as more than space and a commodity, something to be owned, exploited, and profited from [204]. There is a need to acknowledge that Indigenous homelands were never ceded through a treaty in the common law sense; the Indigenous peoples of Canada who signed treaties maintain that they only agreed to share their land [28,29]. Furthermore, from Indigenous peoples’ perspectives, reconciliation must occur among all beings of Creation (i.e., living things and non-living entities) [121,172]. This Indigenous perspective on reconciliation is strikingly different than ‘practical reconciliation’—that is, a deficit model based on the pursuit of statistical equality between Indigenous and non-Indigenous peoples in health, housing, education and employment—a policy championed by the Australian Government [17] and other governments worldwide. As a further matter, the seemingly different goals of Indigenous peoples (e.g., self-government, self-determination, sovereignty, and reconciliation) have all been described as pathways to *mino-mnaamodzawin* (living well or the good life) by the *Anishinaabek* scholar Deborah McGregor [121].

### 4.4. Limitations

The qualitative data collected and analyzed are a snapshot of Canadian Indigenous perspectives prior to the COVID-19 pandemic. Undoubtedly, some specific wellbeing perspectives have changed. However, the general themes reported in the present study should be robust enough that only the specifics would change due to context, not the themes themselves—although there could possibly be additional themes. Further, even though the Indigenous perspectives presented at the local and national levels are for the 2017–2019 time period, the northern Ontarian First Nations’ perspectives presented give perspectives from 2009. However, northern Ontarian First Nations’ perspectives would also have been included in national organizations (e.g., Assembly of First Nations, Native Women’s Association of Canada) submissions and presentations for 2018. For an unknown reason, First Nations in northern Ontario and their regional organizations did not take part in the written submissions or oral presentations in 2018. The oral presentations for 2009 were the most recent data of this type available for northern Ontario, as there were no public hearings for Bill 197, passed in 2020. It should also be communicated that the perspectives given for northern Ontarian First Nations and Indigenous organizations from across Canada were in the context of law-making. In other words, the Indigenous peoples and organizations were not asked the specific questions that Fort Albany First Nations Elders were asked but were, in essence, responding to government bills whose content would significantly affect their wellbeing collectively, regionally (northern Ontario) and nationally (Canada). Nonetheless, we believe that the concordant themes that emerged from the different levels examined in the present study speak to the robustness of the results. Lastly, written submissions and presentations at hearings were impacted due to short notices, insufficient participant funding, page restrictions with respect to written submissions, and presentation time constraints [85,87,89,96,99,211].

## 5. Conclusions

The aims of the present study were to conceptualize wellbeing and determine what was (and is) important for cultural wellbeing from Fort Albany First Nations’ Elders’, and Canadian Indigenous peoples’ perspectives. Overall, Indigenous leadership and organizations viewed wellbeing holistically and conceptualized wellbeing multidimensionally, with physical wellbeing (i.e., health) being one of the many dimensions of wellbeing. Furthermore, although wellbeing at the level of the individual was described, Indigenous perspectives from across Canada also emphasized wellbeing at the familial, community, national, and world scales. This scaling of wellbeing represents a collectivist perspective, not only of the animate but also the inanimate, a commonly held Indigenous belief worldwide. This is why wellbeing was often referred to in the context of interconnectedness, honouring inherent obligations, maintaining (or re-establishing) balance, and harmonious relationships with everything in Creation. Importantly, land was the connecting thread between all types of wellbeing, being a place to practice cultural traditions (land was described as culture onto itself), reassert (or establish) one’s Indigenous identity, find solace, and pass on Indigenous knowledge and language. Land, in this sense, does not refer to the small-circumscribed reserve lands but to the Indigenous homelands of the particular Indigenous group; this is so because treaties were made with Indigenous peoples in Canada on a nation-to-nation basis. Understandably, the level of wellbeing is most often discussed at the cultural level by regional and national Indigenous leadership and organizations, as these people and organizations are representative of broader interests. Thus, non-Indigenous governments in Canada must take a more holistic-and-collectivist viewpoint as a starting point in all interactions with Canadian Indigenous peoples and look at wellbeing at the cultural level. This is especially pertinent because of Canada’s sordid history of past assimilative efforts (e.g., cultural assimilation through residential schools and the ‘sixties scoop’ legal assimilation through enfranchisement, blood quantum requirements) [34,204]. There needs to be a new beginning with land and cultural wellbeing at the forefront. Perhaps, the Government of Canada can move away from its colonial path of assimilation [204] towards reconciliation if Indigenous perspectives of reconciliation are accounted for and respected.

Although there is great cultural diversity among Indigenous nations with understandings of wellbeing specific to each culture, there are general understandings (or values) of wellbeing that are commonly held [154]. Thus, it is not surprising that even in acknowledging the great cultural diversity among Canadian Indigenous nations, four concordant themes were identified regionally and nationally with respect to what was important for cultural wellbeing: land and water, sustainability, and inherent obligations; being on the land, and indigenous languages and knowledge systems; sustainable development; and meaningful involvement in decision-making, and free, prior, and informed consent. However, it must be emphasized that there is no Canadian pan-Indigenous culture or homogeneous perspective of cultural wellbeing because each Indigenous nation has a unique set of relationships and understandings in the context of their homeland [126]. Furthermore, each Indigenous nation’s historical experiences with development differ, and future development on specific Indigenous homelands will also differ. Nonetheless, the centrality of Indigenous homelands to cultural wellbeing was a concordant value found across Canada, with Indigenous leadership and organizations mentioning that they were not against development but that the development must be sustainable from their perspectives. If development is not sustainable from their perspectives, then environmental assimilation—a colonial process—will continue to occur in Indigenous homelands. Environmental assimilation is said to happen when environmental integrity is impacted by colonial development to the point whereby the environment can no longer support Indigenous culture and activities [34,204]. Expectedly, the valued processes identified by regional and national Indigenous organizations important for cultural wellbeing included meaningful participation in the legislative decision-making process, and the inclusion of the free, prior, and informed consent process. In these ways, Indigenous peoples could better safeguard their interests, especially in the context of land, culture, and development. In Canada, the fundamental conflict between colonizers and Indigenous peoples has typically been about the land [136,212,213]. Clearly, it is of fundamental importance that the non-Indigenous governments of Canada acknowledge Indigenous perspectives from across Canada on what is important for their cultural wellbeing—including specificities for each Indigenous homeland—in order to move forward policy on the road towards reconciliation [207,214,215,216].

## Data Availability

Not applicable.

## Supplementary Materials

The following supporting information can be downloaded at: https://www.mdpi.com/article/10.3390/ijerph20176656/s1. Supplemental tables with respect to breadth of coverage, Canadian Indigenous perspectives on what is important for cultural wellbeing (or the valued components of cultural wellbeing). Refs. [217,218,219,220,221,222,223,224,225,226,227,228,229,230,231,232,233,234,235,236,237,238,239,240,241,242,243,244,245] are cited in Supplementary Materials.
