# Supplementary material for: What Is Wellbeing, and What Is Important for Wellbeing? Indigenous Voices from across Canada
Source: Int J Environ Res Public Health. 2023 Aug 26;20(17):6656. doi: 10.3390/ijerph20176656 (PMC10487260; doi:10.3390/ijerph20176656)
Supplement: Supplementary file 1 [file ijerph-20-06656-s001.zip › ijerph-2455044-supplemental materials.pdf]

International Journal of *Environmental Research and Public Health*

MDPI

Supplementary Material:

Table S1. The breadth of First Nations' perspectives from northern Ontario with respect to public hearings for Bill 173 (*Mining Amendment Act, 2009*), and Bill 191 (*Far North Act, 2010*). (note – there were no public hearings for the Ontario *Environmental Assessment Act, 2020*, contained in the omnibus bill entitled the *COVID-19 Economic Recovery Act, 2020*).

First Nations Organization	Notes
Attawapiskat First Nation	An Omushkego Cree First Nation located on the Attawapiskat River near the western James Bay coast.
Chapleau Cree First Nation	*"In 1989, the Chapleau Cree First Nation negotiated with the Federal and Provincial governments and the local municipality for land to establish a permanent community. Chapleau Cree First Nation is currently situated on the Fox Lake Reserve. Since its conception many band members have returned to their community."* [1]
Constance Lake First Nation	*"The Kenogami Watershed…flows north of Kenogami Lake…The majority of this watershed the people of Constance Lake First Nation had made their territory for many years. Constance Lake First Nation members had gathered in an area called Mamawmatawa (where four rivers meet), also known as the English River Reserve."* [2]
Fort Albany First Nation	An Omushkego Cree First Nation located on the Albany River near the western James Bay coast.
Kashechewan First Nation	An Omushkego Cree First Nation located on the Albany River near the western James Bay coast.
Kitchenuhmaykoosib Inninuwug	*"Located approximately 377 miles north of Thunder Bay, Ontario, Canada."* [3]
Matawa First Nations Tribal Council	*"Matawa provides advisory services/programs to nine member Ojibway and Cree First Nations (eight First Nations in James Bay Treaty No. 9) and one First Nation in the Robinson-Huron Treaty area (though they are not a signatory to this Treaty). We are committed to quality assurance and are responsive to our communities' needs."* [4]
Moose Cree First Nation	An Omushkego Cree First Nation located on the Moose River near the western James Bay coast.
MoCreebec Council of the Cree Nation	*"The families of MoCreebec members have been living in the Moose Factory – Moosonee area [of northern Ontario] for generations…trace their ancestry to the [Cree] people along the East Coast of what is now known as James Bay, in the province of what is now Quebec…the Moose Factory [Ontario] Cree of Quebec…formed an association in 1980, which they named MoCreebec." [5]*
Mushkegowuk Tribal Council	*"Composed of eight northern Ontarian First Nations: "We are committed to providing responsible and accountable political leadership. The Mushkegowuk Council is dedicated to providing quality equitable and accessible support and advisory services to respond to and meet the social, economic, cultural, educational, spiritual, and political needs of first nations, thereby improving the quality of life of our people."* [6]

Nishnawbe Aski Nation	*"Nishnawbe Aski Nation (NAN) was established in 1973…NAN territory encompasses James Bay Treaty No. 9 and the Ontario portion of Treaty No. 5, a landmass covering two-thirds of the Province of Ontario, spanning 210,000 square miles. Our people traditionally speak Cree and Algonquin in the east, OjiCree in the west, and Ojibway in the central south area. NAN represents 49 First Nations with a total population (on and off-reserve) of approximately 45,000 people grouped by Tribal Council. Six of our member Nations are not affiliated with a specific Tribal Council."* [7]
Wahgoshig First Nation	*"Near Matheson Ontario …the Wahgoshig First Nation reserve encompasses 19, 239 acres; the north end meets the south shore of Abitibi Lake, which divides Northeastern Ontario from North Western Quebec…The Lake Abitibi Algonquins, historically one band, are now composed of two communities. Wahgoshig (Ontario) and Pikogan (Quebec)."* [8]
Weenusk First Nation	A Cree First Nation located on the Winisk River near the western Hudson Bay coast.
Whitewater Lake First Nation	*"Community of Whitewater Lake is located…250 km N of Thunder Bay, on shore of Whitewater Lake and in centre of Wabakimi Provincial Park."* [9]

Table S2. The breadth of Canadian Indigenous perspectives from written submissions and oral presentations with respect to Bill C-69 (*An Act to enact the Impact Assessment Act and the Canadian Energy Regulator Act, to amend the Navigation Protection Act and to make consequential amendments to other Acts, 2019*).

First Nations Organization	Notes
Algonquins of Ontario	*"Algonquins have lived in present-day Ontario for thousands of years before Europeans arrived. Today, the AOO are comprised of ten Algonquin communities… The Algonquins of Ontario claim includes an area of 9 million acres within the watersheds of the Kichisippi (Ottawa River) and the Mattawa River in Ontario, an unceded territory that covers most of eastern Ontario."* [10]
Assembly of First Nations	*"The Assembly of First Nations (AFN) is the national, political organization of First Nation governments and their citizens, including those living on and off reserve. Every Chief in Canada is entitled to be a member of the Assembly, and the National Chief is elected by the Chiefs in Canada, who in turn are elected by their citizens. The AFN has 634 member nations within its assembly. The role and function of the AFN is to serve as a nationally delegated forum for determining and harmonizing effective, collective and co-operative measures on any subject matter that the First Nations delegate for review, study, response or action, and to advance the aspirations of First Nations."* [11] (p. 4)
Assembly of First Nations Quebec-Labrador	*"Created in 1985, the Assembly of First Nations Quebec-Labrador (AFNQL) is the meeting place of the leaders of 43 communities of eleven First Nations in Quebec and Labrador."* [12] (p. 2)

Athabasca Chipewyan First Nation	*"ACFN are a Dené speaking people. Our primary community is located in Fort Chipewyan, Alberta, but our territory stretches across north-eastern Alberta, Saskatchewan, Manitoba, Northwest Territories and Nunavut. We are signatories to Treaty 8."* [13] (p. 1)
Atlantic Policy Congress of First Nations' Chiefs Secretariat	*"APC of First Nations' Chiefs Secretariat was federally incorporated in 1995 and is a policy research and advocacy Secretariat for 30 Mi'kmaq, Maliseet, Passamaquoddy and Innu Chiefs, Nations and Communities… APC member communities hold Aboriginal title in the Atlantic Provinces and the Gaspé Peninsula. They are all either signatories of Peace and Friendship Treaties 1725-1779 which did not surrender land or water, or they have never signed Treaties with the Crown. APC is governed by a board of directors comprised of the Chiefs."* [14] (p. 1)
British Columbia Assembly of First Nations	*"The BCAFN, as an advocacy organization holds to its mandates from First Nations Chiefs in BC."* [15] (p. 2)
Coastal First Nations	*"A unique alliance of nine BC First Nations, creating jobs for the future and protecting the Great Bear Rainforest."* [16]
Cold Lake First Nations Lands and Resources, Consultation Department	*"Treaty No. 6 Indigenous Nation in Alberta."* [17] (p. 1)
Dene Nation	*"The Dene Nation is a mosaic of different Indigenous groups, including Treaty 8 and Treaty 11 Nations, self-governing Nations, Nations with land claim agreements, and Nations who are currently in a comprehensive claims negotiation process. Self-determination in this region is widespread, and many public Indigenous governments and co-management authorities have been established through modern land claim and self-government agreements."* [18] (p. 1).
Dene Tha First Nation	*"Dene Tha' have lived on our lands in northwestern Alberta, northeastern British Columbia and the southern Northwest Territories since time immemorial. We have just under 3,000 members* [19] (p. 1 of cover letter)
Duncan's First Nation	*"The Duncan's First Nation (DFN) is comprised of Beaver, Cree and Mohawk people…adherent to Treaty#8…two parcels of reserve lands on the east and west bank of the Peace River in Alberta…Our traditional territory takes in large portions of north-western Alberta and north-eastern British Columbia."* [20] (p. 1)
Federation of Sovereign Indigenous Nations	*"The Federation of Sovereign Indian Nations represents 74 First Nations in Saskatchewan. The Federation is committed to honouring the spirit and intent of the Treaties, as well as the promotion, protection and implementation of the Treaty promises that were made more than a century ago."* [21]
File Hills Qu'Appelle Tribal Council	*"FHQTC is the political and service organization for the* Qu'Appelle Agency *and the* File Hills Agency, *delivering programs and services to the 11 First Nations in the Treaty Four Territory."* [22]
First Nations Fisheries Council	*"FNFC is comprised of First Nations delegates from across British Columbia and works with our members to address common issues and concerns with respect to fisheries and aquatic resources."* [23] (p. 1)
Fort McKay First Nation	*"Fort McKay First Nation (Fort McKay) is a First Nation1 community located in northeast Alberta. Fort McKay is an adherent to Treaty 8 and has nearly 830 members of Cree and Dene descent."* [24] (p. 1)

Grand Council of the Crees (Eeyou Istchee)/Cree Nation Government	*"The Grand Council of the Crees (Eeyou Istchee) and Cree Nation Government represent the coming together of the executive and administrative branches of [10] Cree government[s]. We work to promote and protect the interests of the Eeyouch living in eastern James Bay and south-eastern Hudson Bay [of QC]."* [25]
Inuvialuit Regional Corporation and Inuvialuit Game Council	*"Established in 1984 to manage the settlement outlined in the Inuvialuit Final Agreement (IFA). Inuvialuit Regional Corporation (IRC) represents the collective Inuvialuit interests in dealings with governments and the world at large. IRC's goal is to continually improve the economic, social and cultural well-being of the Inuvialuit through implementation of the IFA and by all other available means. Inuvialuit beneficiaries directly control IRC and its subsidiaries through a democratic process of elected directors from each of the six Community Corporations."* [26]
Kebaowek and Wolf Lake First Nations	*"The Algonquin Nation is made up of eleven distinct communities in all. Nine are located in Quebec and two are in Ontario. The Algonquin Nation, which includes our two communities, has never given up aboriginal title to its traditional territory. This includes all the lands and waters within the Kitchisibi or Ottawa River watershed on both sides of the Ontario-Quebec border."* [27] (p. 1)
Lower Fraser Fisheries Alliance	*"LFFA is a voice for First Nations of the Lower Fraser River on matters related to fish and aquatic resources and works with the 30 independent First Nations communities from Tsawwassen to Yale, British Columbia. The LFFA works collaboratively and holistically to support the management and sustainability of First Nation fisheries and supports cultural and spiritual traditions for future generations. The mission of the LFFA is to promote and support the management of a robust and expanding fishery for the First Nations of the Lower Fraser River."* [28] (p. 1)
Makivik Corporation	*"Makivik, which in Inuktitut means "To Rise Up," is a fitting name for an organization mandated to protect the rights [of ~12,000 Inuit], interests and financial compensation provided by the 1975 James Bay and Northern Quebec Agreement, the first comprehensive Inuit land claim in Canada, and the more recent offshore Nunavik Inuit Land Claim Agreement that came into effect in 2008. The Corporation's distinct mandates ranges from owning and operating large profitable business enterprises and generating jobs; to social economic development, improved housing conditions, to protection of the Inuit language and culture and the natural environment."* [29]
Manitoba Metis Federation	*"The Manitoba Metis Federation ('MMF') is the democratically elected government of the Metis Nation's Manitoba Metis Community and is duly authorized by the citizens of the Manitoba Metis Community for the purposes of dealing with Manitoba Metis rights, claims, and interests, including negotiating accommodations and conducting consultations… Currently, there are approximately 60,965 adult Citizens of the Manitoba Metis Community registered to vote in the next MMF election."* [30] (pp. 1-2)
Metis Nation British Columbia	*"Metis Nation British Columbia (MNBC) is recognized by the provincial and federal governments and the Metis National Council as the official governing organization in the province of British Columbia, representing more than 17,000 provincially registered Metis citizen."* [31] (p. 3)
Metis Nation of Ontario	*"Represents 20,000 regional rights-bearing Metis citizens, twenty-nine Chartered Metis Community Councils throughout Ontario and an elected provincial governing body known as the Provisional Council of the Metis Nation of Ontario."* [32] (p. 1)

Mi'gmawe'l Tplu'taqnn	*"Mi'gmawe'l Tplu'taqnn was founded…2015 to advance, protect and implement the Aboriginal and Treaty Rights of its member communities, which are the nine Mi'gmaq communities in New Brunswick. In this process, Mi'gmawe'l Tplu'taqnn represents eight of our member communities."* [33] (p. 2).
Mikisew Cree First Nation	*"Mikisew Cree First Nation is the largest First Nation in the oil sands region. The heart of our traditional territory is the Peace Athabasca Delta in Wood Buffalo National Park and the lands and waters around the Athabasca River."* [34] (p. 1)
Mohawk Council of Kahnawake	*"The Mohawk Council of Kahnawa:ke is the governing body for the Mohawk Territory of Kahnawa:ke [in QC]. It is the organization that provides governmental, administrative, and operational services to the community of Kahnawa:ke."* [35] (p. 11)
Musqueam Indian Band	*"Musqueam Indian Band is an Indigenous nation with traditional, ancestral, and unceded territory located in what is now the city of Vancouver and surrounding areas in the province of British Columbia. Musqueam retains Aboriginal rights and title across all lands and waters within Musqueam's core territory, as described in the 1976 Musqueam Declaration."* [36] (p. 7)
Native Women's Association of Canada	*"The Native Women's Association of Canada (NWAC) is founded on the collective goal to enhance, promote, and foster the social, economic, cultural and political well-being of First Nations, Métis and Inuit women. NWAC is an aggregate of thirteen Native women's organizations from across Canada and was incorporated as a non-profit organization in 1974. Our mission is to help empower women by being involved in developing and changing legislation which affects them, and by involving them in the development and delivery of programs promoting equal opportunity for indigenous women."* [37] (p. 10)
Nunatsiavut Government	*"Nunatsiavut Government represents the Labrador Inuit, and is a regional self-government under the Labrador Inuit Land Claims Agreement."* [38] (p 1 of the cover letter)
Nuu-chah-nulth Tribal Council	*"Nuu-chah-nulth Tribal Council (NTC) is a not-for-profit society that provides a wide variety of services and supports to fourteen Nuu-chah-nulth First Nations with approximately 10,000 members."* [39]
Okanagan Nation Alliance	*"The Okanagan Nation Alliance (ONA) was formed in 1981 as the inaugural First Nations government in the Okanagan [BC] which represents the 8 member communities…Each community is represented through the Chiefs Executive Council (CEC) by their Chief or Chairman."* [40]
Peguis First Nation	*"Peguis First Nation located in the Interlake Region in the Province of Manitoba…Ojibway and some Cree decent…12000 strong in membership…Peguis First Nation has one of the biggest traditional territories in central Canada and our history shows that our migration route was from the Red River Region Just North of Winnipeg, MB to Red Lake Minnesota, to Garden River Ont. and Peguis has family trees connecting them to these communities…Signatory to the signing of Treaty 1."* [41] (p. 2)
Skeena Fisheries Commission	*"Established in 1990…by the five First Nations in the [Skeena] watershed—the Tsimshian, Gitxsan, Gitanyow, Wet'suwet'en, and Lake Babine Nations…Our membership has evolved over the years, and currently includes the Gitxsan, Gitanyow, and Wet'suwet'en Nations…We support our Member Nations' implementation of their*

	inherent rights and responsibilities to conserve, protect, manage, and sustainably harvest fish and aquatic resources within their territories. We also support effective participation and meaningful engagement in fisheries policy and management processes that impact Skeena fish populations and habitat." [42]
The First Nations Major Projects Coalition	*"Established in 2015, the Coalition is comprised of 40 First Nations in BC with partnerships growing with First Nations elsewhere in Canada (Alberta, Manitoba, Ontario). The Coalition's mandate is technical and not political. The First Nations-led organization is designed to support communities at arriving at informed decisions concerning major project development."* [43] (p. 1)
Tsleil-Waututh Nation	*"Tsleil-Waututh are the 'People of the Inlet' and a distinct Coast Salish nation whose territory includes Burrard Inlet in the lower mainland of British Columbia."* [44] (p. 1)
Wolastoqey Nation in New Brunswick	*"The Wolastoqey Nation in New Brunswick (the "WNNB") consists of Kingsclear First Nation, Madawaska Maliseet First Nation, Oromocto First Nation, St. Mary's First Nation and Tobique First Nation. WNNB provides technical advice to Wolastoqey leadership on consultation and resource development matters that relate to the implementation and exercise of Wolastoqey constitutionally protected rights. WNNB aspires to protect and promote traditional lands, rights, ceremony, cultural practices, and language."* [45] (p. 1)

Table S3. Northern Ontarian First Nations' perspectives on what is important for cultural wellbeing (or the valued components of cultural wellbeing).

Themes	Representative Quotes
Land and Water	*"When we're talking about the land, the people are connected to the land. First Nations people are stewards of the land; it's part of us."* (Chief Keeter Corston of Chapleau Cree First Nation) [46] (p. 955)
	"I just wanted to say that the elders here, the Nishnawbe Aski…our job is to give advice to our chiefs…I just want to come to the point that our land is not for sale. It is not for sale. We want to keep that." (Gregory Koostachin, Nishnawbe Aski Nation Elder) [47] (p. 958)
Sustaining the Environment	*"Our concepts of preserving Mother Nature…We are one with the land, we depend on it to feed our families, and we have thousands of years of intergenerational experience with how to live in harmony with the land and preserve it, not destroy it in a few years."* (Sam McKay, Councilor for Kitchenuhmaykoosib Inninuwug) [48] (p. 912)
	"[W]e protect our lands. They've been protected for thousands of years." (Chief David Babin of Wahgoshig First Nation) [49] (p. 955)
	"A lot of people say it's our last frontier. What the government instead should be doing is congratulating all of the First Nations and NAN territories for keeping the land in its natural state: the way it is. We have not contaminated and harmed our land." (Chief George Hunter of Weenusk First Nation) [50] (p. 956)

	"We've got to think about tomorrow. We've got to think about our kids, our children who are coming. What are we going to leave them? Are they going to live on nothing?" (Chief David Babin of Wahgoshig First Nation) [49] (p. 955)
Honouring Inherent obligations	*"When you talk about jurisdiction, that there are only two ways you can have jurisdiction: You can inherit it—one way—or you get delegated it. The province [of Ontario] and the feds got delegated by the Queen of England. First Nations here, we inherited it from our Creator. These are the things that you have to learn and to understand as MPPs [Members of Provincial Parliament] or MPs [Federal Member of Parliament]."* (Chief Andrew Solomon of Fort Albany First Nation) [51] (p. 954)
	"We are the north. It is our land, and we govern and protect by our inherent right given to us by the Creator. We have protected and governed the lands for thousands of years. The legacy of our care is that our use has been next to invisible. To you, the lands look untouched. They aren't. They've just been touched by the Anishnawbe in accordance with Anishnawbe laws and customs. That's why the lands are in the condition they are in. We will continue to protect and govern the lands for future generations." (Frank Beardy, Special Envoy for Nishnawbe Aski Nation) [52] (p. 953)
	"I want to make clear that these bills [173 and 191], if passed, will not be recognized as valid laws in our territory. We hold aboriginal rights, including title, to our unceded and un-surrendered traditional territory. We have never signed on to Treaty 9 or surrendered our lands, and as such, the province has no jurisdiction over our traditional territory…We have our own consultation and accommodation policy, a policy of exploration, and we are in the process of developing a lands and resources policy." (Chief Theresa Hall of Attawapiskat First Nation) [53] (p. 981)
Being on-the-land	*"As remote [fly-in] aboriginal communities who intensively use our traditional territories, we are the ones who will suffer the most serious impacts [of mining]."* (Sam McKay, Councilor for Kitchenuhmaykoosib Inninuwug) [48] (p. 912)
	"You talk about [protected] parks…We can't even go hunting; we can't even go fishing. If we build a little cabin, we're all thrown in jail. For what? You took us off our land. You took us away from our home so you can develop industry." (Chief David Babin of Wahgoshig First Nation) [49] (p. 955)
	"It was stated earlier that we're nomadic people, and the treaty gave us something really different. A lot of people are displaced. The young people today don't know who they are. Their identity is lost. So you have a high rate of suicide. Those are the symptoms of the treaties and the policy-making of the governments." (Chief Andrew Solomon of Fort Albany First Nation) [51] (p. 953)
Indigenous languages and knowledge systems	*"Nishnawbe Aski First Nations hold inherent First Nations aboriginal and treaty rights…It is not an exaggeration to say we are the north. The pathways of that place are filled with our stories and our history and are governed by our laws and customs. To this day, only First Nations people live there."* (Grand Chief Stan Beardy of Nishnawbe Aski Nation) [52] (p. 828)
	"I've got grandkids. I want a future for them. I want to teach them about the land that I grew up in, where my grandparents taught me." (Chief Jonathon Solomon of Kashechewan First Nation) [54] (p. 954)

Table S4. Northern Ontarian First Nations' perspectives on what processes are important for cultural wellbeing.

Themes	Representative Quotes
Sustainable Development	*"We are not against [sustainable] development. All we're saying is, we continue to live in poverty while the province gets wealthier and richer."* (Chief Jonathon Solomon of Kashechewan First Nation) [54] (p. 954)
	"You polluted all south of 50. You cut every tree; you've ruined it. Species are at risk…and still you want more. You want to go north of 50 now; you want to go north there because you've ruined it here. I've warned the northern chiefs [of unsustainable development]. I live south of the 50th parallel, and I've seen the behaviour…These people are here to protect their homelands that belong to them. It doesn't belong to Ontario. The land was never surrendered." (Chief Keeter Corston of Chapleau Cree First Nation) [46] (pp. 955-956)
Meaningful Participation in the Decision-making Process	*"They want to create a super-park on our land—225,000 square kilometres. We will not have a say…We become nobody. Respect is a two-way street, and if the government of Ontario is willing to come forward to respect our inherent and treaty rights, I would welcome that with open arms. But at this point in time, I cannot and will not succumb to the pressures that we are put under."* (Chief Jonathon Solomon of Kashechewan First Nation) [54] (p. 954)
	"The bill requires 225,000 square kilometres. The First Nations' traditional territory is protected and off limits with respect to development. This was made without consultation with us or any First Nation in the Far North, and means that there will be instances when a First Nation wishes to support development on their land but is barred from doing so due to it being protected land." (Chief Theresa Hall of Attawapiskat First Nation) [53] (p. 982)
	"Protected zones have hindered the development of First Nations…limits any potential revenue generation to them at a future date." (Chief Arthur Moore of Constance Lake First Nation) [55] (p. 964)
	"The Ontario government to me has never dealt in good faith, and I remember this Northern Table: I followed it quite closely. For once the First Nations thought that maybe there was some small chance that the government was going to be honourable and truthful, and that didn't happen, as usual." (Chief Keeter Corston of Chapleau Cree First Nation) [46] (p. 955)
	"Matawa First Nations, including chiefs, counsellors and community members, participated in several Mining Amendment Act [Bill 173] forums…Regrettably, most of those recommendations were not included in the new Mining Act amendments. This is not a question of consultation but rather, were our people listened to? Consultation is only as good as the accommodation that arises." (Chief Arthur Moore of Constance Lake First Nation, spoke on behalf of the Matawa First Nations Tribal Council) [55] (p. 963)
	"In our discussions with the government of Ontario, we have always stressed that we come to you in a spirit of good faith… When the revisions of the Mining Act came about, we again said, 'We want to participate and be a part of the changes that we would like to see within the Mining Act.' We went in the tent, so to speak, with the government officials to work on the changes that we would like to see that reflect on the concerns and issues of our people. Every time we came

	to a clause that we would like to see enacted as law, we were told, 'We will deal with that at the policy level.' We did not enter into these discussions to influence policy. We went into these discussions to influence what the wording of the law should be. A few months after that, we went in the tent to deal with the land use planning act [Bill 191]. Again, the same thing happened." (Frank Beardy, Special Envoy of Nishnawbe Aski Nation) [52] (pp. 958-960)
	"We started out with land use planning being First-Nations-led. By the time we got to the legislation, that had been watered down to 'significant involvement' for First Nations, as determined by the minister at her unilateral discretion…In Bill 191 [Far North Act, 2010], Ontario holds all the cards, makes all the important decisions, sets out all the rules and tries to placate First Nations with the illusion of participation and control. That is why Bill 191 has been rejected and why the Nishnawbe Aski Nation First Nations will exercise their jurisdiction and control over their lands as they have a right to do." (Frank Beardy, Special Envoy of Nishnawbe Aski Nation) [52] (p. 952)
Free, Prior, and Informed Consent	*"The current version of Bill 173 [Mining Amendment Act, 2009] does not provide for consent, a key requirement put forward by the First Nations in Ontario… We've told the province from day one that it is the people in our home communities who need to have the discussion and need to have input into the process. That has fallen on deaf ears…resource developers and the province and government can consult with what they deem to be consultation until they're blue in the face, and they can accommodate us and accommodate certain things [but]…at the end of the day, there has to be consent."* (Grand Chief Stan Louttit of Mushkegowuk Tribal Council) [56] (pp. 984-986)
	"The scope of the constitutional duty to consult, accommodate and sometimes seek First Nations consent depends on the nature of the government policy or legislative proposal…Bill 191 is a fundamental threat to…NAN First Nations…it does two things. First, it gives Ontario control of land use planning in the far north area…it imposes an interconnected protected area of a super-park of at least 225,000 square kilometres…This will ensure that NAN First Nations will remain part of the Third World forever…Each First Nation, including Whitewater Lake First Nation, has a veto over profound impact as a matter of Canadian constitutional law." (Chief Arlene Slipperjack of Whitewater Lake First Nation) [57] (p. 948-986)

Table S5. Canadian Indigenous perspectives on what is important for cultural wellbeing (or the valued components of cultural wellbeing).

Themes	Representative Quotes
Land and Water	*"We always identify ourselves as to where we're from. That is our connection to the land and the water, and that's our jurisdiction. That's who we are. We're part of our ancestors."* (Tsleil-Waututh Nation) [44] (p. 22)
	"First Nation, Inuit and Métis Canadians depend on [the environment] for sustenance, culture and spirituality. The economies and cultures of Indigenous peoples is inseparably woven with their lands and natural resources...Land lies at

	the heart of social, cultural, spiritual, political, and economic life for Indigenous women. The survival of Indigenous communities, their well-being and empowerment depend on their relationship to the land and waters, and the environmental abilities of Indigenous women to transmit their knowledge." (Native Women's Association of Canada) [37] (pp. 2-7)
	"For First Nations, the free and unencumbered use of the waterways in their territories is critical to their cultures and ability to exercise a range of s.35 rights, and for other important purposes." (Assembly of First Nations) [11] (p. 26)
	"Navigation is integral to the exercise of all other rights of Indigenous peoples throughout their territories, from harvesting and spiritual practices to resource governance." (First Nations Fisheries Council) [23] (p. 9)
Sustaining the Environment	*"[P]ractices of sustainability that we have practiced for thousands of years on our territories. Indigenous institutions are essential for future prosperity and participation in evolving targets for sustainability, biodiversity and climate change under agreements to which Canada is signatory."* (Kebaowek and Wolf Lake First Nations) [27] (pp. 9-10)
	"The LFFA works collaboratively and holistically to support the management and sustainability of First Nation fisheries and supports cultural and spiritual traditions for future generations." (Lower Fraser Fisheries Alliance) [28](p. 1)
	"[Governments] need to understand the Indigenous world view prior to any major project being given the green light." (Regional Chief T. Teegee of the British Columbia Assembly of First Nations) [58] (p. 17)
Honouring Inherent obligations	*"Our traditional perspective and world view that all aspects of the natural world, of which people are part, need to be respected and cared for."* (Tribal Chief Edmund Bellegarde of Files Hills Qu'Appelle Tribal Council) [59] (p. 2)
	"Our decision-making authority over our traditional territory is inherent. The outstanding step toward recognition of our rights is to recognize that inherent decision-making power in federal statutes." (Coastal First Nations) [16] (p. 5)
	"Tsleil-Waututh holds a sacred, legal obligation and responsibility to our ancestors, current, and future generations to protect, defend, and steward the water, land, air, and resources of our territory. Our stewardship obligation includes the need to maintain and restore conditions that provide the environmental, cultural, spiritual, and economic foundation for our nation and community to thrive. The Tsleil-Waututh Nation does this through actively asserting and exercising its stewardship and governance rights." (Chief Maureen Thomas of Tsleil-Waututh Nation) [60] (p. 1)
Being on-the-land	*"The James Bay and Northern Quebec Agreement…Crees are active participants in the orderly development of the resources in Eeyou Istchee so as to safeguard their hunting, fishing, and trapping rights, as detailed in section 24 of the treaty."* (Bill Namagoose, Executive Director, Grand Council of the Crees (Eeyou Istchee)) [61] (p. 3)<br>*"Any changes to the environment will directly affect Indigenous women's and girls' health, wellbeing, and identity…Indigenous women's relationship to the environment is inseparable from their cultural knowledge, teachings, and identity. Their unique identities are often shaped by time spent, knowledge learned, and gifts given from the land. Environmental degradation and extractive industries influence their ability to be able to carry out their responsibilities to the land or engage in land-based activities integral to their cultural identities. Violence on the land often translates directly into violence against Indigenous women and their ability to carry out and transmit culture. Effectively, denying Indigenous*

	women the equal opportunity to self-determination is allowing systemic cultural genocide to progress." (Native Women's Association of Canada) [37](p. 7)
	"Over the past several decades our Traditional Territory has been subjected to waves of successive development that have heavily impacted our lands, waters, fish and animals that we have a relationship and rely upon. The cumulative impact of agriculture, hydro projects, oil and gas, oil sands, mining, forestry and over hunting and fishing have impacted the ecology of our lands and has made it difficult to impossible for our people to meet their livelihood and cultural needs and exercise their rights." (Duncan's First Nation) [20] (p. 1)
	"Kichisippi Pimisi (American eel) is considered sacred to the Algonquin people and has been a central part of our culture for thousands of years…Hydroelectric dams have caused a catastrophic decline [of] this culturally significant species in our traditional watershed…The Lake Sturgeon too is a species culturally significant to the Algonquin…also suffered major decline from dams…Fluctuating water levels and unnatural water flows have significantly impacted fish spawning." (Algonquins of Ontario) [62] (pp. 3-4)
Indigenous languages and knowledge systems	*"Relatively pristine…Moose Lake Reserves were expanded to promote cultural preservation. Cultural preservation and the transmission of traditional knowledge includes but is not limited to hunting, fishing, trapping and gathering on those Reserves and the surrounding lands."* (Fort McKay First Nation) [24] (p. 4)
	"It is not appropriate to assume that all Indigenous peoples of Canada have a common understanding of cultures, traditional knowledge or perspectives in particular areas." (Coastal First Nations) [16] (p. 11)

Table S6. Canadian Indigenous perspectives on what processes are important for cultural wellbeing.

Themes	Representative Quotes
Sustainable Development	*"Fort McKay is not opposed to oil sands development…among the most proactive of First Nations with respect to oil sands development. Working in the oil sands sector has brought to the First Nation and its members opportunity, economic self-sufficiency, stability, and prosperity that are inaccessible to many First Nations people across the country, but…Fort McKay is also surrounded by oil sands development…Working with industry to advance shared objectives requires mutual respect and an acknowledgement that section 35 grants to all First Nations the right to continue a way of life."* (Chief Jim Boucher of Fort McKay First Nation) [63] (p. 18)
	"Dene Tha' is not opposed to development. We know the importance of economic opportunities, and strive to obtain those for our own members. However, development needs to be sustainable and, consistent with the United Nations' Declaration on the Rights of Indigenous Peoples (UNDRIP), consent-based." (Dene Tha First Nation) [19] (p. 2)

	"Often Aboriginal people are cast in the role of folks adamantly opposed all the time to development…certain values that it wants to protect and uphold around the environment…[but] If there isn't any investment in Canada in major projects . . . [the result] plays out in our community in high levels of unemployment, poor housing . . . a lack of infrastructure improvement and maintenance in our communities . . . we want to make sure that they [Indigenous children] enjoy the same living standards . . . along with other Canadians." (Chief and Indigenous Co-Chair E. Crey of the Indigenous Advisory and Monitoring Committee for the Trans Mountain Pipelines and Marine Shipping) [64] (p. 25)
Meaningful Participation in the Decision-making Process	*"As the Indigenous peoples do not participate in a decision…a proponent can impact their physical or cultural heritage or their health, social or economic conditions without their consent and without committing an offence [legally]."* (Nunatsiavut Government) [65] (p. 3)
	"In the spirit of reconciliation, the Minister should not be left with the discretion whether or not to engage in an agreement with Indigenous authorities; the decision to engage should be left with the Indigenous authorities themselves." (Athabasca Chipewyan First Nation) [13] (p. 4)
	"Reconciliation…confirmation from an affected First Nation that its views and concerns have been addressed. First Nations' inherent jurisdiction must be recognized…when the Government of Canada begins respecting and fulfilling commitments made in treaties, both historic and modern…essential to enable us to move forward together in a good way." (Interim Regional Chief Kluane Adamek of the Yukon Region, Assembly of First Nations) [66] (p. 16)
Free, Prior, and Informed Consent	*"Respect the free, prior, and informed consent standard throughout a full and honourable joint process."* (Assembly of First Nations) [11] (p. 4)
	"[There should be] adoption of the United Nations Declaration on the Rights of Indigenous Peoples; including free, prior and informed consent of indigenous Peoples." (Assembly of First Nations Quebec-Labrador) [12] (p. 6)
